# The Skin Microbiome: Current Landscape and Future Opportunities

**DOI:** 10.3390/ijms24043950

**Published:** 2023-02-16

**Authors:** Paisleigh Smythe, Holly N. Wilkinson

**Affiliations:** 1 Centre for Biomedicine, Hull York Medical School, University of Hull, Hull HU6 7RX, UK; 2 Skin Research Centre, Hull York Medical School, University of York, York YO10 5DD, UK

**Keywords:** microbiome, skin, ageing, senescence, wound healing, infection, antimicrobials, metagenomics, skin models

## Abstract

Our skin is the largest organ of the body, serving as an important barrier against the harsh extrinsic environment. Alongside preventing desiccation, chemical damage and hypothermia, this barrier protects the body from invading pathogens through a sophisticated innate immune response and co-adapted consortium of commensal microorganisms, collectively termed the microbiota. These microorganisms inhabit distinct biogeographical regions dictated by skin physiology. Thus, it follows that perturbations to normal skin homeostasis, as occurs with ageing, diabetes and skin disease, can cause microbial dysbiosis and increase infection risk. In this review, we discuss emerging concepts in skin microbiome research, highlighting pertinent links between skin ageing, the microbiome and cutaneous repair. Moreover, we address gaps in current knowledge and highlight key areas requiring further exploration. Future advances in this field could revolutionise the way we treat microbial dysbiosis associated with skin ageing and other pathologies.

## 1. Introduction

The skin is a highly dynamic organ composed of a range of cell types and structures that work together to preserve the cutaneous barrier and counter external challenges. The main layers are the epidermis and dermis, with underlying subcutaneous adipose tissue providing cushioning and energy reserves for the body [1]. The skin also houses appendages, such as hair follicles and glands, which are involved in many homeostatic functions, from thermoregulation to wound repair [2,3,4]. Sebaceous glands secrete non-polar lipids to prevent water loss [5], while antimicrobial peptides (AMPs) excreted from sweat glands limit the growth of pathogenic organisms [6,7]. Although the dermis preserves the structural integrity of the skin, the epidermis is the primary defence barrier maintaining direct contact with the extrinsic environment. This barrier comprises multiple biological, structural and chemical components crucial for preventing internal infection. However, perturbations to the cutaneous barrier, as occurs with skin ageing, pathology and injury, can cause microbial dysbiosis and increase infection risk [8,9,10]. Thus, this review will summarise existing knowledge of the dynamic interactions between the skin and its resident microbes, highlighting key disparities and future opportunities in this exciting field.

## 2. The Skin Barrier

The epidermis includes multiple heterogeneous layers, each performing a specialised role to preserve the skin barrier. The basal layer of the epidermis contains keratinocytes with stem-cell-like characteristics, which are attached to an underlying specialised matrix, the basement membrane [11]. A large proportion of the basal keratinocytes remain affixed to the basement membrane, but a subset of daughter cells progress through the epidermal layers via asymmetric mitosis [12]. This crucial mechanism enables self-renewal of the epidermis in a process known as terminal differentiation [13]. Daughter keratinocytes first enter the stratum spinosum, forming a layer of polyhedral-shaped cells joined together by desmosomes, intracellular junctions that mediate cell–cell adhesion and reinforce the epidermis against physical trauma [14]. Above the stratum spinosum is the stratum granulosum, a layer of flattened keratinocytes that form cytoplasmic keratohyalin granules to crosslink keratin filaments and create the water-impermeable barrier [15]. Finally, keratinocytes enter the external tier of the epidermis, the stratum corneum, where they release lysosomal enzymes that degrade their intracellular components [16]. This results in cells that are terminally differentiated, enucleated and tightly crosslinked to strengthen the skin barrier. As the stratum corneum is constantly shed and replaced every four weeks, the cycle of stratification is continuous and must be tightly controlled to prevent breaches to the skin surface [17,18].

**Figure 1:**
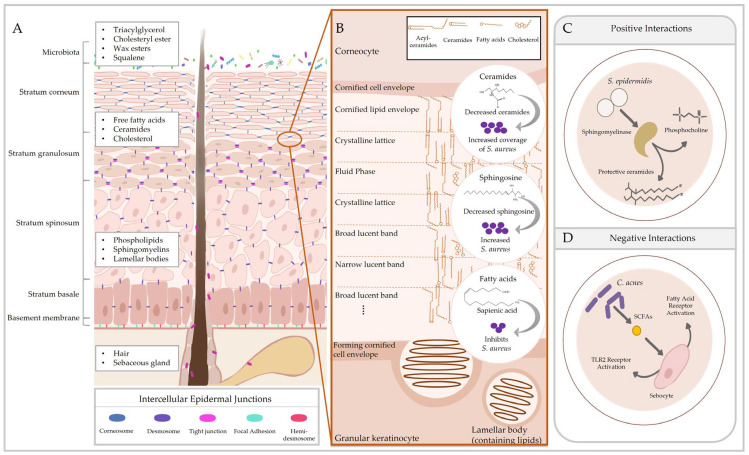
Cutaneous lipids preserve the epidermal permeability barrier and influence host–microbe interactions. The epidermis consists of multiple layers of phenotypically distinct keratinocytes that contribute to stratification. (**A**) Keratinocytes in each tier exhibit different adhesion structures responsible for maintaining skin integrity. Epidermal lipids are observed in the differentiating layers of the epidermis, while sebaceous lipids are secreted from sebaceous glands. (**B**) Molecular arrangement of the main stratum corneum lipids from [51] and effects of those lipids on *Staphylococcus aureus* colonisation [52,53,54]. (**C**) Skin microbiota also produce metabolites to utilise cutaneous lipids, generating products that contribute to barrier homeostasis (e.g., sphingomyelinase in *Staphylococcus epidermidis* [55]). (**D**) In pathological conditions, these interactions may negatively affect skin physiology (e.g., *Cutibacterium acnes* enhancing inflammation [56]). SCFA = short-chain fatty acids.

Like other barriers of the body, the cutaneous barrier consists of microbial, immune, chemical and physical components [19]. However, unlike other epithelia, the skin exhibits an epidermal permeability barrier comprising the stratum corneum and a complex of tight junctions, adhesion proteins and cytoskeletal networks. Together, these structures prevent passive water loss from the body and protect against harmful chemical and biological agents [20]. This is apparent in investigations of epidermal function, where deficiencies in skin barrier proteins result in improper barrier formation, increased transepidermal water loss [21], reduced epidermal proliferation and differentiation [22,23] and skin barrier disorders [22,24]. Corneocytes of the stratum corneum are cemented together by a densely packed three-dimensional lipid matrix composed of ceramides, cholesterol and free fatty acids [5,25]. This matrix is formed from sebum produced by sebaceous glands, but also contains epidermal lipids and AMPs released from lamellar bodies of keratinocytes in the stratum granulosum [26]. The lipid layer of the stratum corneum safeguards the skin from desiccation by forming an impermeable barrier and provides a substrate for a range of microbial interactions (summarised in Figure 1). In particularly thick areas of the skin, such as the foot, there is an additional layer between the stratum granulosum and stratum corneum, the stratum lucidum, which acts as another impenetrable barrier to water [27].

The generation of free fatty acids on the surface of the skin creates a low-pH environment (pH 4–6) essential for barrier homeostasis [28]. An acidic skin pH is crucial for the activity of epidermal enzymes required for lipid processing and regulating cohesion proteins, thus preserving the stratum corneum and maintaining hydration levels [29,30]. Low skin pH also conserves the commensal skin microflora, which act as a first-line defence against pathogens through direct competition, influencing cutaneous immunity and supporting barrier homeostasis [31,32,33,34]. A range of immune cell subsets exist in the skin, secreting a plethora of cytokines and chemokines to modulate host response [35]. Indeed, skin colonisation with commensal bacteria shapes immunity through the activation of pattern recognition receptors, with the distinct activation signature dictating skin physiology. Activation of immune response pathways by commensal Staphylococcal spp. strengthens the immune barrier to prevent pathogenic infection [33], while commensals also trigger activation of Toll-like receptors (TLRs) to bolster immunity and accelerate wound repair [36]. Moreover, knockdown of certain pattern recognition receptors, such as NOD2, causes microbial dysbiosis and delayed wound healing [37], further demonstrating the importance of effective immune sensing in regulating cutaneous microbial colonisation. 

Keratinocytes likewise possess important innate immune functions, expressing TLRs and other immunomodulatory proteins (e.g., NOD2; [38]) that recognise pathogenic substances (pathogen-associated molecular patterns; [39]). Immune response pathways are activated in a TLR-specific manner, causing the release of cytokines, chemokines and AMPs to attract circulating immune cells. In addition to TLRs, keratinocytes constitutively express certain AMPs, such as HBD1, while others are induced in response to injury and infection [34,40,41]. Sebum lipids similarly contribute to immune surveillance, as many free fatty acids upregulate AMPs and cytokines in sebaceous glands [42,43,44] and display direct antibacterial properties [45]. Moreover, sebaceous-gland-rich sites of the skin harbour higher levels of AMPs (e.g., S100A7, DEFB4B and LCN2), chemokines and barrier genes than sebaceous-poor sites [46], suggesting that skin exhibits distinct immunological topography that could play a role in dictating the microbial composition of different skin sites.

TLRs are also required for barrier function as injury induces TLR3 to initiate inflammation [36], while activation of TLR2 elevates the expression of tight junction proteins (e.g., claudins and occludin) to enhance human skin barrier repair [47]. As TLRs are activated by microbial molecular signals, these findings indicate that resident microbes may be imperative for barrier maintenance and repair. This is certainly the case with other xenobiotic receptors; for example, mice deficient in the aryl hydrocarbon receptor exhibit an impaired skin barrier and are more susceptible to pathogenic colonisation with *S. aureus*. Further, colonisation of skin with a conglomerate of human skin commensals reduces transepidermal water loss and increases barrier gene expression in gnotobiotic mice [48]. Moreover, in humans, skin barrier perturbations (e.g., in atopic dermatitis) are associated with a lower abundance of protective skin commensals [8,49,50] and perturbations in TLR expression [47]. Despite these pertinent studies elucidating major roles of pattern recognition receptors in skin homeostasis, the vastly diverse landscape of host–microbe interactions remains largely unexplored due to the complexity of these interactions and the challenges associated with microbiome research (discussed in Section 8). 

## 3. Skin Topography and the Microbiome

**Figure 2:**
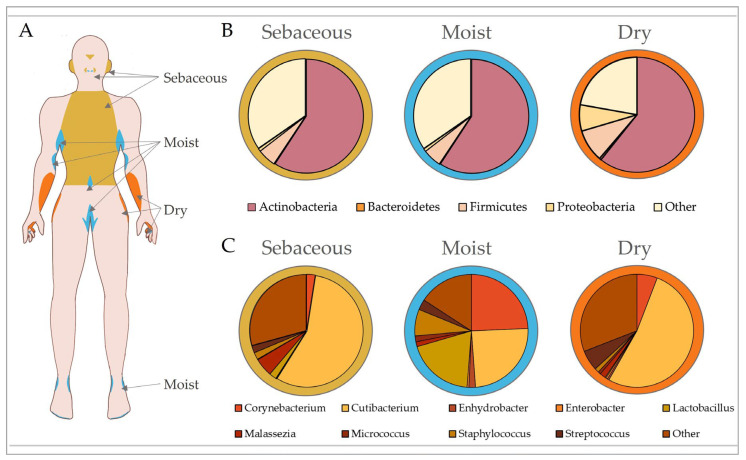
Microbial composition of the skin is dictated by topography. Skin exhibits biogeographically distinct regions that are generally categorised according to their unique physiological characteristics into sebaceous, moist and dry sites (**A**). These regions exhibit different microbial community structures influenced by skin pH, temperature, sebum content and moisture levels. Microbiota are separated by phylum according to site-specific variation in (**B**), with top contributing genera shown in (**C**). In healthy adults, sebaceous (e.g., torso, glabella and retroauricular crease) and dry (e.g., volar forearm and hypothenar palm) sites are predominantly colonised by *Cutibacterium*, while moist sites (e.g., antecubital fossa, inguinal crease and axillary crease) show equally high abundances of *Corynebacterium*, *Cutibacterium* and *Staphylococcus*. Data analysed from Oh et al. [94].

Maintenance of the cutaneous barrier is clearly critical to prevent pathogenic infection, with traditional skin microbiology focussing on prevention and treatment of infection by well-known pathobionts for this very reason [57]. Yet, the advent of gut microbiome research has now shifted our focus towards understanding the dynamic interactions that occur between the skin and its largely symbiotic community of bacterial, fungal, viral and Archaean inhabitants, collectively deemed the microbiota [58]. Our skin is second only to the gut in terms of bacterial density, with an approximate density of 10^4^ to 10^6^ bacteria per square centimetre and over 200 genera characterised [59]. The skin is home to 18 phyla, with 4 dominant ones: Actinobacteria (51.8%), Firmicutes (24.4%), Proteobacteria (16.5%) and Bacteroidetes (6.3%) [60]. Although skin microbiome research is still in its infancy, especially compared to that of the gut, pertinent studies have identified key roles for skin microbiota in maintaining homeostasis. These include providing nutrients (vitamin and amino acid synthesis [61,62]), inhibiting pathogenic growth [63,64], priming our immune system to differentiate between commensals and pathogens [65,66], and regulating epidermal differentiation [67]. A large proportion of the microbiome consists of resident microbes that are generally stable, but there is a smaller percentage of transient microbes that can opportunistically colonise niches when the skin is compromised [10]. Similar to the gut [68,69], the skin houses microbial communities that inhabit spatially distinct regions dictated by cutaneous topography (summarised in Figure 2; [70,71]), and while site-specific microbial composition is largely conserved, it can be affected by a range of other individual attributes, such as age, ethnicity, genetics, climate and skincare [71,72,73,74,75,76]. Skin diseases can also alter the microbiome and often present in a site-specific manner [10], indicating that exploration into the microbial habitation of ecological niches may provide significant insight into a range of skin pathologies.

The varied biogeography of the skin provides an unprecedented opportunity to explore how biological niches affect microbial composition. While the gut is rich in micro- and macronutrients that promote the growth of beneficial bacteria [77], the skin is a hostile environment with limited resources available. Thus, skin microbiota are specialised to utilise the chemical milieu of the stratum corneum, sweat and sebaceous glands. It is therefore not surprising that certain bacteria thrive in different skin regions, where there are varying levels of ultraviolet radiation (UVR) exposure, temperature, moisture, sebum content, oxygen availability and pH [78]. The skin microbiome shows the greatest conservation at higher taxonomic levels [56], and although bacterial communities group by skin type (sebaceous, dry and moist sites), fungi tend to segregate by body region alone [79]. 

The following seminal publications reveal the topographic and temporal distribution of skin microbiota, yet a major drawback of these studies is that they provide low taxonomic resolution, rarely classifying microbes beyond genus level. This is important given that the skin harbours a plethora of microbial species with broad phylogenetic representation [80,81]. Individual strains of bacteria also show differential associations with skin health and pathology, a prime example being species within the genus *Staphylococcus* [82,83,84]. Consequently, future studies should shift their focus to understanding strain-level temporospatial diversity of the skin.

Sebaceous (oily) sites (e.g., the torso, back and face) are highly acidic due to the abundance of free fatty acids [28]. These regions are predominantly inhabited by bacteria that can metabolically utilise sebum and tolerate low pH, such as *Cutibacterium* (formerly *Propionibacterium* [60]). As *Cutibacterium* spp. require anaerobic growth conditions, they are found in the pilosebaceous units of sebum-rich areas, where they produce lipases to convert sebum triglycerides into short-chain fatty acids, including propionic acid [85]. This is further supported by the fact that facial sebum levels directly correlate with *Cutibacterium* prevalence [86]. *Staphylococcus* is the second most predominant genus in the sebaceous skin microbiome. Staphyloccocci, such as *Staphylococcus aureus* and *Staphylococcus epidermis*, are tolerant of the acidic pH found in oily skin and produce lipases to utilise the lipid-rich substrate of these sites [32,87,88]. Interestingly, the retroauricular crease is a sebaceous site with low phylotype richness that tends to remain temporally stable due to the prevalence of *Cutibacterium* [78,89].

Moist sites (e.g., antecubital fossa, inguinal crease and popliteal fossa) are areas with a higher temperature and humidity, and a variety of hair follicles and glands [90]. The moist niche provides a myriad of nutrients, such as salts, sterols, esters and lipids, enabling the growth of *Staphylococcus* and *Corynebacterium* [60]. Corynebacteria are dominant colonisers of warm and moist environments [91], while the halotolerant *Staphylococci* are found at high density in moist, salt-rich sites such as sweat glands [92]. Although the microbiota of moist skin remains relatively stable, shifts in diversity can be observed between individuals [93,94]. By contrast, dry sites (e.g., hypothenar palm and volar forearm) exhibit high microbial diversity and low temporal stability [60,70,94]. Interestingly, different studies display variability in the dominant bacteria reported in dry skin, which may be influenced by bacterial biomass and temporal stability. For example, Flavobacteriales and β-Proteobacteria were dominant in Grice et al. [60], but *Cutibacterium* showed the highest contribution to dry sites in Oh et al. [70].

Although the microbiota consists of a wide range of microorganisms, most microbiome research has focussed on characterising the topography and function of bacteria, with less consideration given to our fungal, viral and Archean inhabitants due to their generally lower abundance [79,95]. Nevertheless, there have been some advancements in profiling the skin’s fungal microbiome (or mycobiome). As with bacteria, fungi reside within each skin niche, with community structure linked to skin region and age-related changes (reviewed in [96]). Culture-based methods initially identified *Malassezia*, *Rhodotorula*, *Debaromyces*, *Cryptococcus* and *Candida* as commensal skin flora [97,98,99]. These findings have since been extended using amplicon sequencing to reveal site-specific variation in fungal communities, where there is a general predominance of *Malassezia*. Additionally, the foot mycobiome is vastly more diverse than that of other body sites and includes *Malassezia*, *Aspergillus*, *Cryptococcus*, *Rhodotorula* and *Epicoccum* [79]. Further investigations are required to fully characterise fungal residency on the skin and provide insight into the interactions between fungal, bacterial and host communities.

## 4. The Skin Microbiome throughout the Life Course

Understanding normal skin physiology and microbial interactions enables greater appreciation of homeostatic mechanisms but also provides insight into how perturbations to normal cutaneous function are associated with disease. Indeed, skin topography, function and microbial composition are not only dictated by body site but can be influenced by a range of endogenous (e.g., genetics, age and gender) and environmental (e.g., lifestyle, climate, air pollution and use of cosmetics) factors [78,100,101]. The inherent high complexity and variability of the skin microbiome makes it challenging to delineate its role in specific physiological processes [102]. Despite this, several pertinent studies have shed light on how the skin microbiota change throughout the life course [103,104,105], and how perturbations in microbial composition are associated with skin disease and ageing [106,107,108].

Even though first microbial contact is thought to begin in utero [109], microbial colonisation rapidly expands at birth with exposure to the atmospheric environment [110]. Initial microbial colonisation is limited to a particular set of bacterial taxa from the birth canal, but is dictated by delivery route, as babies born via caesarean section exhibit microbiome profiles that more closely mimic the mother’s skin (dominated by *Staphylococcus*, *Corynebacterium* and *Cutibacterium*) than vagina (dominated by *Lactobacillus* and *Prevotella* [111]). Birth mode also affects the relative abundance of specific fungal spp., with vaginal births resulting in higher levels of *Candida albicans* [112]. However, by six weeks of age, microbial composition cannot be distinguished by delivery route [113]. Additionally, the vaginal microbiome changes throughout gestation, with pregnant mothers displaying lower bacterial diversity and higher contribution of *Lactobacillus* compared to non-pregnant women [114,115] and women postpartum [116]. 

Initial skin microbial colonisation is responsible for shaping the successional transition of the microbial ecosystem into adulthood. This first contact, alongside pre-programmed mechanisms, is crucial for the development of infant immunity [117], which preferentially develops tolerance to skin commensals [66,118]. It has been postulated that disruption of native microbial transfer, as observed with C-section, heightens the risk of type 1 diabetes due to links between the microbiota and immune system [119]. However, metanalyses assessing these risks have found mixed results [120,121]. By contrast, disrupting microbiota during pregnancy (e.g., with antibiotic use) may contribute to disease risk in offspring. This is certainly true in vivo, as vancomycin given to pregnant does markedly shifts gut microbial composition and increases asthma severity in murine offspring [122,123]. Thus, an appreciation of microbiome development in early life provides insight into the associations between microbial dysbiosis and pathology, proposing that the skin microbiota could be harnessed to improve health.

The microbiota of new-borns is undifferentiated across different body regions [111,113], but site-specific changes begin to occur within three months of age, with microbial diversity stabilising over the first year of life [113,124]. The diversity of the skin microbiome continues to adapt throughout childhood [125] and adolescence [126] and is shaped by the changing microenvironment of different skin sites during sexual maturation [127]. For example, both *Cutibacterium acnes* and *S. epidermidis* increase in a site-specific manner upon sexual maturity in males and females, while the lipophilic *C. acnes* and *Malassezia restricta* positively correlate with higher concentrations of certain sex hormones in female children (estrone, 17β-estradiol and testosterone [127]). These alterations are likely influenced by the effects of hormones on the skin, such as androgens increasing sebaceous gland activity [128]. Moreover, certain *C. acnes* strains are predicted to produce excessive porphyrin, which can cause skin inflammation and contribute to development of acne vulgaris [129]. These findings may perhaps shed light on why certain microbiome-linked skin pathologies are dependent on life stage.

In adults, the microbiota of the skin remains relatively stable, but as discussed previously, is highly dictated by the skin’s topography [94]. It has even been suggested that the observed stability of the adult skin microbiome could be used to predict an individual’s chronological age within four years [130]. Adult skin displays higher levels of *Cutibacterium* and *Corynebacterium*, while young children show a dominance of Gammaproteobacteria and Streptococcaceae at multiple sites [131]. These shifts in microbial composition coincide with physical changes in the skin, as the skin of infants is thinner, more alkaline and has a higher cell turnover rate compared to adults [132,133,134]. It is therefore not surprising that naturally aged skin, featuring many physiological and structural modifications, exhibits drastic compositional alterations in the skin microbiome [76,104,105,134]. 

## 5. Skin Ageing and the Microbiome

**Figure 3:**
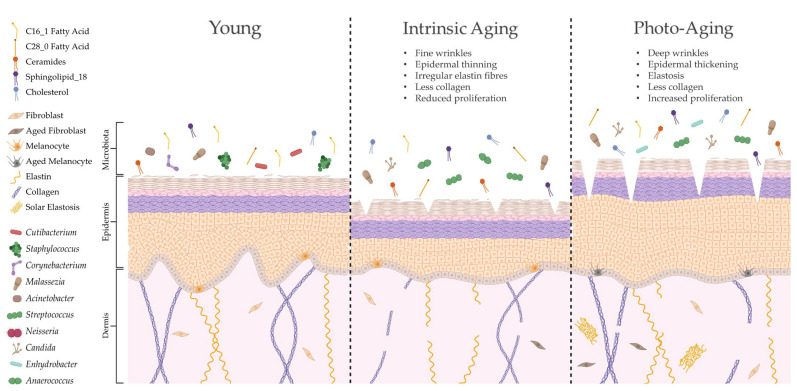
Ageing alters skin structure, function and microbial colonisation. Intrinsic ageing and photoageing cause differential alterations to cutaneous architecture and physiology, resulting in marked shifts in microbial composition [86,106]. Indeed, this altered skin microbiome may be shaped by specific modifications in lipid composition [9], which could further contribute to age-related cutaneous pathology. Created with biorender.com (accessed on 11 January 2023).

The skin is unique compared to other organs in that skin ageing is dictated by intrinsic and extrinsic factors. Sun-protected sites of the body, such as the buttocks, largely undergo intrinsic ageing processes influenced by genetic, metabolic and hormonal changes (e.g., reduction in 17 β-estradiol; [135]). Intrinsically aged skin is characterised by reduced sebaceous gland function, decreased blood flow and degradation of collagenous and fibrous extracellular matrices (ECMs), leading to atrophy, reduced lipid content, xerosis and fine lines [136,137]. By contrast, extrinsic ageing is triggered by environmental factors but is predominantly dictated by exposure to UVR [138]. Therefore, extrinsically aged skin is commonly found in sun-exposed sites of the body, such as the face and hands, and is depicted by telangiectasia, hyperpigmentation, deep wrinkles and a leathery appearance [139]. Both intrinsically and extrinsically aged skin have a higher pH, less hydration and reduced expression of tight junction proteins compared to young adult skin, but unlike intrinsic ageing, photoageing causes elevated proliferation and increased sebum [136,140]. Certain skin constituents that are altered with age (e.g., lipids) are also linked to microbial surveillance and skin barrier homeostasis [42,141]; hence, it is unsurprising that aged skin features marked dysbiosis in microbial composition (summarised in Figure 3). 

Intrinsic and extrinsic ageing trigger differential structural and functional alterations to the skin, yet both processes cause similar age-related changes in species richness compared to young adult skin [106]. As intrinsic ageing underpins both types of ageing, these results suggest that microbial diversity is highly dictated by intrinsic ageing mechanisms. This has been corroborated in other studies in women, with the within-sample microbial diversity of aged skin being significantly higher than that in young adult skin and most significant in sun-protected sites [9,104,108,141]. Overall abundance of bacteria increases with age, but this is not directly proportional as certain bacteria become more dominant (e.g., *Corynebacterium*) while others decline in number (e.g., *Cutibacterium* and *Lactobacillus* [9,104,108,141]) in a site-independent manner. Certain genera are also altered by body site, such as higher abundance of *Streptococcus* and lower abundance of *Staphylococcus* on the buttocks [9]. Age-related mycobiome shifts include reduced *M. restricta* and greater abundance of other fungal spp. on cheeks [104].

Several studies have performed correlative analyses to attempt to link the age-associated alterations in the skin microbiome to the structural and physiological changes that occur with intrinsic and extrinsic skin ageing [9,86,104,141]. Indeed, Howard et al. [9] demonstrated that ageing decreases the facial sebaceous gland area and increases the number of ceramides, lipids and natural moisturising factors, which positively and negatively correlate with specific bacterial genera. Moreover, Kim et al. [104] revealed that the dominant metabolic pathways of aged skin bacteria were linked to fatty acid degradation, antibiotic biosynthesis and bacterial motility. However, it is difficult to directly link the observed correlations between microbial dysbiosis and skin ageing without undertaking mechanistic investigations. Many of these studies are simultaneously constrained by utilising small cohorts, one gender and one ethnic group, making it difficult to generalise findings. Moreover, these studies often do not identify microbes beyond genus level, yet it is known that individual strains of bacteria are linked to age-associated skin pathology [82,83,84,142]. Future investigations should therefore aim to characterise age-related changes in the microbiome using more sophisticated techniques, appropriate study cohorts and selecting skin sites that enable delineations between intrinsic and extrinsic ageing.

A handful of pertinent investigations have begun to elucidate how age-related alterations in bacteria can affect host fitness; it has been suggested that age-specific dominant microbes can influence immunity and inflammation [108,143] and may regulate the intrinsic ageing process (reviewed in [144]). Notably, preventing the age-related decline in gut compartmentalisation limits microbial dysbiosis and extends lifespan [145]. In the skin, age-related changes in microbial community structure could drive pathogenic colonisation, contributing to many of the deleterious effects described above. In fact, *Cutibacterium*, which is reduced in aged skin, produces free fatty acids and thiopeptide antibiotics to suppress the growth of methicillin-resistant *S. aureus* and group A *Streptococcus* [146,147]. Other commensal bacteria, such as *Bacillus* and coagulase negative *Staphylococcus*, can also produce antimicrobials or induce the skin’s innate immune response to prevent pathogenic growth [33,34,148]. Nevertheless, our knowledge of age-related shifts in microbial taxonomy remains enigmatic, making it difficult to extrapolate the associated functional consequences for the skin.

## 6. Cellular Ageing and Microbial Dysbiosis

Cellular senescence (cellular ageing) is an area of research gaining considerable traction for its role in intrinsic skin ageing and potential for therapeutic targeting [149]. Cellular senescence is a mechanism whereby cells undergo transient or permanent cessation of proliferative capacity in response to intrinsic and/or extrinsic stressors linked to the ageing process, such as DNA damage, oxidative stress, mitochondrial dysfunction, inflammation and telomere shortening [150]. Senescent cells remain viable yet have significant metabolic and genetic alterations compared to their non-senescent counterparts, including the reorganisation of chromatin [151], increased lysosomal activity [152,153] and activation of a DNA damage response [154]. The central dogma is that senescence evolved to suppress tumours in young organisms by preventing neoplastic growth [155]. However, senescent cells accumulate with increasing physiological age, contributing to a range of age-related diseases by virtue of their functional perturbations and unique secretome (or senescence-associated secretory phenotype, SASP [156]. The SASP includes a range of secreted cellular products (e.g., growth factors, cytokines, chemokines, proteases and lipids [156,157,158]) that alter the tissue microenvironment in a context-dependent manner. For example, in wounds, a transient SASP mediated by PDGF-AA enables effective deposition of ECMs during skin repair [159], while a chronic SASP governed by CXCR2 delays healing in diabetes [160]. SASP genes are known to be upregulated at the transcriptional level by NF-κB and C/EBP β, but other pathways are also involved (e.g., mTOR) [161,162]. Moreover, the SASP can induce senescence in a paracrine manner, thus exacerbating tissue destruction and ageing phenotypes [163,164,165].

Aged skin is characterised by high levels of senescence in the epidermis, dermis, skin appendages and subcutaneous tissue [166,167,168,169]. Dermal fibroblasts from intrinsically aged skin also produce a secretome including canonical SASP factors (e.g., IL-1B, MIF and PAI-2 [156]), and proteins that may be unique to skin ageing [170]. Senescent cell accumulation in aged tissues is partially driven by age-related decline in the innate and adaptive immune systems, which are required to effectively clear senescent cells [171]. In addition, senescent cells are able to evade immune clearance by expressing antigens to inhibit natural killer cell activation and avoid T-cell recognition [172,173]. More recently, it has even been suggested that senescent fibroblasts can evade immune detection by secreting specific lysophospholipids [158]. Tissues with high levels of senescence display reduced functionality, increased inflammation and degradation of the ECM, due in part to the excessive production of matrix metalloproteinases (MMPSs) [174,175,176]. The fragmentation of ECM components, such as collagen and elastin, is clearly apparent in aged dermis exhibiting high levels of senescence and MMPs [177,178,179]. Multiple authors have demonstrated that catalase activity is reduced in intrinsic and photoaged skin, leading to elevated oxidative stress, MMP1 expression and collagen fragmentation [180,181]. This fragmentation of collagen even stimulates MMP1 production in fibroblasts cultured in vitro [181] to cause further ECM breakdown.

Though few direct links have been made between senescent cell accumulation and the physical characteristics of skin ageing, it is established that senescent fibroblasts cause hallmark signs of skin ageing, such as reduced epidermal thickness and impaired barrier formation in vitro [182] and in 3D skin equivalent models [183,184]. We know far less about the role of epidermal senescence in skin ageing, with most studies in this area focussing on the effects of UV exposure on senescence induction in keratinocytes and fibroblasts [185,186,187]. Interestingly, epidermal senescence elevates skin barrier permeability [188], which could be instigated by disruption of tight junctions [189,190], loss of structural proteins [191] and aquaporins [192] and decreased production of the enzymes responsible for sphingolipid synthesis [193]. In the epidermis, melanocytes are the primary cell type expressing the canonical senescence marker, p16ink4a, during skin ageing [166,194], and they contribute to skin atrophy in 3D skin equivalents via a CXCR3-governed SASP [164]. Indeed, these studies reveal that senescence drives skin barrier defects, which may impact microbial dysbiosis during skin ageing.

While microbiome changes in the skin correlate with age-associated alterations in cutaneous physiology [9,104], functional links between the skin microbiota and cellular ageing remain to be investigated. Most pertinent studies linking the microbiota to cellular senescence involve the gut, demonstrating that microbial dysbiosis enhances senescence and contributes to age-related disease states [195,196]. Moreover, ablation of senescent cells using senescence-targeted drugs (senolytics) alters gut microbial composition and ameliorates ageing pathology in mice [197,198], whereas a healthy gut microbiome is directly linked to healthy ageing in humans [199]. Notably, commensal *Cutibacterium* spp., which are depleted in aged skin, produce antioxidants capable of protecting the skin against cellular ageing [200], while genotoxins produced by pathogenic bacteria readily induce senescence in T cells [201]. Collectively, these findings suggest an important therapeutic link between the microbiome, cellular senescence and age-related pathology that could extend to the skin. Future investigations should therefore begin to explore this area, perhaps by identifying the microbial drivers of age-related changes in the skin using longitudinal metagenomic characterisation, followed by mechanistic assessment of the role of these “age-related” bacteria on the skin barrier and dermal matrix. Moreover, studies should be undertaken to explore how manipulation of senescence (e.g., by using murine ageing models and senolytics) may alter the skin microbiome and affect skin function. This will also have important implications for skin integrity and wound repair, which is discussed below. 

## 7. Wound Pathology, Ageing and Infection 

Breaches of the skin barrier expose subcutaneous tissue to microbial colonisation. In young, healthy individuals, closure of this barrier occurs rapidly via an orchestrated series of cellular events [202]. However, in ageing and related pathologies, skin repair is severely delayed [203], increasing risk of infection and the development of chronic, non-healing wounds [204,205,206]. Chronic wounds are a major socioeconomic burden, costing UK and US healthcare providers billions to treat annually [207,208]; thus, there is an urgent unmet need to fully elucidate the factors contributing to age-related delayed skin healing to develop new and effective therapies. In normal healing, a short-term, transient senescence is required to enable effective deposition of new connective tissue [159] and prevent excessive fibrosis [209]. However, in poor-healing wounds, there are high levels of infection and inflammation that prolong senescence and promote tissue breakdown [160,210,211,212]. Further, we have demonstrated that senescence in diabetic wounds is directly linked to healing outcome, as the ablation of senescence significantly accelerates wound repair in mice [160], while others show that levels of senescence may predict healing in human chronic wounds [212].

Aged skin is more susceptible to infection, in part because the immune system becomes senescent [213]. Altered microbial composition could also increase infection risk following injury, due to the higher numbers of pathobionts [82,214] and lower levels of commensals known to protect against pathogens by modulating host immunity and inhibiting bacterial virulence mechanisms [33,146,215]. This extends to elderly patients with diabetes who are at high risk of developing diabetic foot ulcers. These patients exhibit reduced expression of AMPs, such as RNAse 7 [216], and an abundance of virulent strains of *S. aureus* on their feet, both of which can independently increase infection risk [217]. Interestingly, oxidative stress (a key inducer of senescence) may additionally promote microbial dysbiosis in ageing and diabetes [218].

Recalcitrant wound infection is a key contributor to poor healing in the elderly that is exacerbated by high levels of antimicrobial resistance (AMR) and the presence of bacterial biofilms [219,220,221,222]. Vascular insufficiency is another important factor linked to infection in chronic wounds, decreasing the influx of immune cells and reducing efficacy of systemic antibiotics [223]. In addition, vasculopathy and neuropathy mask hallmark signs of infection, such as pain and erythema, making it difficult to diagnose infection at an early intervention stage [224]. Despite these risks, no studies to date have correlated changes in the skin microbiome with vasculopathy or neuropathy. 

In normal healing, interactions between commensal bacteria (e.g., *S. epidermidis*) and host immunity are necessary to mediate effective repair [225], yet impaired host response leads to microbial dysbiosis and delayed healing in mice [226]. Many reports have measured the microbial composition of chronic wounds [227,228] and correlated microbial composition to healing outcome [82,229,230,231,232]. However, these investigations rarely mechanistically demonstrate the functional links between microbial dysbiosis and healing. Due to the compromised skin barrier, wounds show a higher contribution of opportunistic pathogens, such as *S. aureus*, and lower amounts of commensal *Staphylococcus* and *Corynebacterium*, than intact skin [82,231,232]. Non-healing chronic wounds also exhibit overrepresentation of facultative anaerobes, such as *Enterobacter* and *Proteus*, compared to wounds that heal within six months [232]. Moreover, the recent observations that specific strains of *S. aureus* are associated with poor healing outcomes [82] and infection [83], whereas others bolster host response and skin regeneration [233,234], highlight the need for subspecies-level profiling to elucidate the microbial drivers of poor healing in the elderly.

## 8. Challenges in Skin Microbiome Research 

### 8.1. Microbial Identification

Traditional microbial research focusses on understanding the interactions between the host and individual pathogenic organisms in isolation. However, the emerging evidence that the microbiome plays important roles in health and disease has created a need to (1) accurately identify the microbes that inhabit biological niches and (2) enable collective profiling of native microbial communities [235]. Nevertheless, developing identification techniques that can suitably cover the breadth and depth of our microbial ecosystems is a major challenge in microbiome research. Traditionally, microbial communities have been explored primarily using culture-based methods, which select for species capable of growing in artificial laboratory conditions and greatly underrepresent the diversity of the native microbial environment [236,237]. Indeed, culture-based assays can underestimate the bioburden of diabetic foot ulcers by up to 26 bacterial species and fail to identify *S. aureus* in over 50% of samples [238]. Various 16S techniques have also been found to be advantageous over culture-based protocols for analysing wound swabs and biopsies, identifying around 50% more bacterial species, including obligate and facultative anaerobes [238]. Despite the poor resolution provided by culture-based practices, they are still necessary to enable full exploration of host–bacteria interactions, and their combination with system-level approaches, such as omics technologies [239] and computer modelling [240,241], can provide crucial insight into microbial diversity and function. 

Over thirty years ago, Woese and Fox [242] pioneered community non-culture-based profiling to circumvent some of the challenges associated with traditional culture techniques. They demonstrated that targeted amplification of highly conserved 16S ribosomal RNA (rRNA) genes could be utilised for taxonomic identification because they include hypervariable regions that can be used to classify bacteria based on sequence homology [243]. The 16S rRNA sequencing approach remains the most widely utilised method in microbiome research to date, but is not without its disadvantages. Biases can be introduced by the PCR amplification methods and sequencing platforms used, resulting in inaccurate estimates of microbial diversity [244,245,246]. Moreover, 16S rRNA sequencing provides limited resolution, being rarely capable of classifying bacteria beyond the genus level. This is an important consideration for skin research, as bacteria within the same genus can stimulate differential effects on the host. For instance, atopic dermatitis patients with a predominance of *S. aureus* exhibit more severe disease than those with a high prevalence of *S. epidermidis*, while disease progression can be linked to one dominant strain of *S. aureus* alone [247].

Early sequencing technologies greatly improved our understanding of microbial diversity but remained incapable of providing full microbial resolution or extrapolating phenotypic information. Development of next-generation sequencing has facilitated more detailed characterisation of the microbial landscape, thus revolutionising skin microbiome research [248]. Next-generation sequencing still utilises targeted amplification methods, such as 16S rRNA for bacteria, and internal transcribed spacer regions of the rRNA cistron for fungi [249], but it also enables metagenomic sequencing of entire genomes in a high-throughput, cost-effective manner [250]. Current metagenomic technologies include shotgun sequencing and newer “third generation” single-molecule long-read sequencing techniques (e.g., Oxford Nanopore and PacBio; comprehensively reviewed in [251]). Long-read sequencing (<10 kilobase pairs), although exhibiting higher error rates than short-read approaches, significantly improves taxonomic classification accuracy [252]. Nevertheless, individual platforms exhibit their own biases in taxa detection, making it difficult to discern true community structure and accurately compare findings of individual studies [253]. It has also been suggested that widely utilised sequencing techniques greatly overestimate species richness and diversity because they do not distinguish between extracellular DNA and DNA from viable bacteria [254]. To circumvent some of these issues, several studies are now adopting an integrated metagenomics approach to utilise multiple platforms, gaining the higher accuracy of short-read Illumina alongside the greater resolution of long-read Nanopore [255]. However, integrating workflows from entirely different platforms remains challenging [256], meaning this approach is not yet widely employed. Biases may even be resolved using mathematical modelling to identify the source of bias in workflows and correct them [253], whilst novel methods are being adopted to enable profiling of live bacterial DNA [254]. 

A major advantage of using metagenomics versus targeted amplification is that it can enable assembly of entire genomes, providing functional characterisation alongside higher taxonomic resolution, which is crucial given that different bacterial strains are associated with skin disease (e.g., *C. acnes* [257,258]). Metagenomic technologies have been utilised in assembling bacterial genomes to comprehensively profile the skin microbiome [259], along with identifying taxonomic and functional profiles of gut microorganisms in obesity [260]. Metagenomic sequencing also permits characterisation of the AMR gene reservoir of skin microbes (e.g., *S. epidermidis* [80]). Emerging single-cell approaches even have the potential to take this technology further, not only facilitating strain-level resolution, but also allowing investigation of the interactions between individual bacteria and colocalised bacteriophages [261]. These technologies will certainly be essential for the future development of targeted therapies to tackle AMR in skin and wound infections.

Despite the obvious advantages of metagenomics for microbiome research, full functional resolution and de novo assembly are frequently restricted to highly abundant strains due to the high read depth coverage required. This is particularly challenging in the skin, for which samples (e.g., swabs) often contain very small bacterial yields with varying microbial mass and high levels of host DNA contamination [262]. There is also a greater computational demand to assemble, map and analyse the data. To begin to address these challenges, statistical packages have been developed to utilise taxonomic and phylogenetic data to predict functional profiles and gene redundancies, and to identify biologically relevant differences between microbial profiles [263,264,265]. In addition, advanced techniques are improving read depth coverage (e.g., adaptive sequencing [266]) and increasing the reproducibility and translatability of microbiome research by standardising pipelines [267,268]. Indeed, combining these advances in microbial metagenomics with other technologies (e.g., RNA-Sequencing [269]) will enable a full system-level understanding of the role of the microbiome in skin health and disease.

### 8.2. Modelling the Skin Microbiome

It is becoming more apparent that the skin microbiome is crucially linked to cutaneous health and pathology [33,108]. Thus, suitable skin microbiome models are required for pre-clinical testing of potential therapies and cosmetics, alongside a better understanding of host–microbial interactions. However, development of suitable cutaneous microbiome models remains challenging due to the complexity and diversity of the microbiota and the dynamic interactions that exist between the host and microbial community [270,271]. Moreover, there is still a paucity of studies utilising skin models for microbiome research, with a large number instead focussing on pathogenic colonisation [272,273,274]. Key considerations in developing realistic microbiome models include providing a stable microbial community that represents normal skin microbiota and using models that faithfully mimic skin structure and physiology.

The simplest microbiome models provide a platform to elucidate fundamental relationships between individual bacteria and monolayers of host cells in vitro [275]. Although these models allow us to delineate specific host–bacterial interactions, such as how *S. aureus* can induce serine protease activity [276] and how *S. epidermidis* can activate epidermal defence in keratinocytes [277], they remain a reductionist approach that cannot recapitulate the complexities of a full microbial ecosystem in living skin. In addition, growing cells (bacterial and mammalian) in artificial culture alters their physiological responses, as cell culture media provides a very different nutritional source to natural skin milieu [278], and culture temperatures are several degrees higher than skin temperature [279]. To circumvent some of the challenges associated with in vitro culture systems, Van Der Krieken et al. developed a stratum corneum model to enable assessment of bacterial communities exposed to a more native substrate [280]. Interestingly, the authors demonstrated that bacterial diversity was maintained for up to seven days following inoculation. Nevertheless, this approach is still limited by the fact that the host component of the model is not viable. 

Three-dimensional human skin equivalents provide a more dynamic way to explore cutaneous physiology, comprising epidermal and dermal structures produced by skin cells. They more closely model natural cellular behaviours by providing a more native ECM and enabling true paracrine signalling between cells [281]. As stratification is an important component of skin physiology and host response, a major aim of developing 3D constructs is to create an epidermal barrier that closely resembles native skin, with differentiated layers and a similar composition of epidermal lipids [282]. These models can be useful to explore pathological host–microbe interactions, such as by demonstrating that reduced filaggrin expression increases *S. aureus* colonisation [283]. Human skin equivalents are also commercially available (reviewed in [284]), providing researchers with highly reproducible models requiring minimal additional resources. Skin equivalent models have even facilitated our understanding of the role of single and mixed communities of bacteria in regulating epidermal proliferation, differentiation and metabolism [271,285].

Despite their advantages, engineered skin constructs often lack more complex aspects of skin structure and physiology, such as glands, appendages, blood vessels and immune cells [202]. In recent years, three-dimensional skin constructs have been developed that harbour key components of innate skin physiology, such as vasculature [286,287], appendages [288] and improved immunocompetence [289,290]. This will not only advance the physiological relevance of these models, but simultaneously presents a more realistic environment with which to study the skin microbiome. For example, sebaceous glands provide a niche for *Cutibacterium*, a highly prevalent member of the native skin microbiota [60,85]. Thus, development of more sophisticated skin models incorporating sebaceous-like structures would greatly facilitate future microbiome studies.

By far, the most translational non-animal laboratory approach is to use living human skin, because it provides the advantage of preserving the intrinsic skin structure and cellular heterogeneity. Native skin maintains some immunological competence by virtue of containing resident immune cells that remain present for the first few days of culture [291,292]. Skin explants can be collected from a variety of body sites and donors spanning different ages, genders, ethnicities and pathological conditions to provide full representation of the human population [293]. Indeed, structural and functional changes in aged skin are associated with alterations in microbial composition [9,106], and ethnicity and gender can differentially alter skin pH [294], a key factor in determining microbial composition. Fresh human skin is an incredibly valuable resource for skin research but is often difficult to access and must be utilised within a specific window of viability. In addition, skin cultured ex vivo lacks a systemic response, which is particularly important when exploring the links between skin immunity and microbiota. A systemic influx of immune cells is also imperative for wound healing [295] and clearing pathogenic infection [296]. Indeed, novel culture methods, such as microfluidics, may circumvent some of these limitations by extending tissue viability ex vivo [297] and improving the immunocompetence of human skin equivalents [298].

Much of our understanding of the function of the microbiome has been derived from in vivo murine models that enable mechanistic insight into host–microbial interactions in complex living systems. In vivo skin microbiome research ranges from elucidating the role of commensal bacteria in skin development and maintenance [299,300] to evaluating how pathogenic dysbiosis leads to skin disease [10,29] and delays repair [37,226]. Murine models are also used to evaluate the efficacy of antimicrobial therapies against specific skin pathogens, such as *S. aureus* [301,302]. It is important to note, however, that there are fundamental differences in skin physiology, immunology and microbiology between mice and humans, which constrains the translatability of murine studies [303,304]. Moreover, there are a range of factors that can sway experimental outcomes, from animal strain and housing conditions to the type of infection model used [305,306,307], while the simultaneous underreporting of animal research protocols makes it difficult to extrapolate findings [308]. Pigs offer a more human-relevant model to assess skin physiology [309], but they are less tractable than rodents, and the porcine skin microbiome is less understood. Thus, future research should focus on developing methods to increase reproducibility and more faithfully represent the microbial diversity and complexity of human skin.

## 9. Future Directions

The rapid expansion of gut microbiome research has provided a conduit to explore the role of microbiota in other physiological systems, including the skin. An in-depth appreciation of the factors that shape the temporospatial distribution of cutaneous microbial communities could offer insight into the role of microbiota in skin ageing and pathology. Indeed, current research is often limited by correlative analysis and low taxonomic resolution; therefore, it is paramount that future investigations utilise cutting-edge sequencing methods and translatable models to provide real-world functional insight into host–microbe interactions in the skin.

Despite our limited understanding of the physiological role of skin microbiota in cutaneous biology, several strategies have been implemented to modulate the microbiome to improve health. This includes the use of antimicrobial products produced by bacteria to treat skin infection [148,310,311], and transplantation of commensal Gram-negative bacteria to ameliorate skin disease [49,312]. Moreover, the use of probiotics and postbiotics shows promise in alleviating ageing pathology [313,314,315] and accelerating wound repair [316,317] in experimental models. It would be fascinating to extend these findings to characterise how bacteria-derived treatments modulate the microbiome, alongside elucidating the potential microbial drivers of skin ageing and senescence-linked wound pathology. Wider adoption of sophisticated metagenomic technologies could facilitate functional characterisation of the microbiome and perhaps even provide a personalised approach to diagnosing and treating conditions underpinned by microbial dysbiosis.

In addition, AMR poses an urgent global healthcare challenge [318]; therefore, it is essential to develop non-antibiotic therapies to treat infection. It is now also appreciated that broad-spectrum antibiotics deplete the resident microbiota, contributing to the growth of AMR organisms [319,320]. Hence, emergent therapies must specifically target pathogens to ameliorate any impact on host commensals. One area that holds promise is the formulation of exogenously engineered bacteriophage-derived products capable of selectively killing specific skin pathogens (e.g., *S. aureus* [301]). However, we still require a greater understanding of the pathogenic landscape of skin, and the physiological mechanisms that promote pathogenicity, to utilise antimicrobials more effectively. Indeed, integrated system-level approaches may enable us to address this by providing a greater understanding of microbial community dynamics and delineating the functional relationships that exist between the skin and microbiota. Emerging computational methods could be utilised to integrate datasets and remove individual bias from different studies [253], therefore offering unprecedented insight into the global microbial landscape of the skin in health and disease. Computational methods may even be used to predict longitudinal progression in conditions underpinned by microbial dysbiosis [241], thus circumventing the need to collect longitudinal samples. Indeed, identification of the bacterial molecular signatures associated with certain disease states could even enable development of new and efficacious therapies for cutaneous pathologies and beyond.

## Data Availability

Three search databases were used (PubMed, Google Scholar and Web of Science) to establish pertinent literature to remove biases associated with individual search engines. Key words were associated with relevant topics where applicable. We did not exclude publications based on date of publication, location of research or journal of publication. We based our assessment solely on research quality (as per the San Francisco Declaration on Research Assessment). We also did not search for specific methodologies (except for Section 8) as these can be indexed inconsistently.

## References

[1] Zwick R.K., Guerrero-Juarez C.F., Horsley V., Plikus M.V. (2018). Anatomical, Physiological, and Functional Diversity of Adipose Tissue. Cell Metab..

[2] Ito M., Liu Y., Yang Z., Nguyen J., Liang F., Morris R.J., Cotsarelis G. (2005). Stem cells in the hair follicle bulge contribute to wound repair but not to homeostasis of the epidermis. Nat. Med..

[3] Lu C.P., Polak L., Rocha A.S., Pasolli H.A., Chen S.C., Sharma N., Blanpain C., Fuchs E. (2012). Identification of stem cell populations in sweat glands and ducts reveals roles in homeostasis and wound repair. Cell.

[4] Rittie L., Sachs D.L., Orringer J.S., Voorhees J.J., Fisher G.J. (2013). Eccrine sweat glands are major contributors to reepithelialization of human wounds. Am. J. Pathol..

[5] Pappas A. (2009). Epidermal surface lipids. Dermatoendocrinology.

[6] Rieg S., Seeber S., Steffen H., Humeny A., Kalbacher H., Stevanovic S., Kimura A., Garbe C., Schittek B. (2006). Generation of multiple stable dermcidin-derived antimicrobial peptides in sweat of different body sites. J. Investig. Dermatol..

[7] Schittek B., Hipfel R., Sauer B., Bauer J., Kalbacher H., Stevanovic S., Schirle M., Schroeder K., Blin N., Meier F. (2001). Dermcidin: A novel human antibiotic peptide secreted by sweat glands. Nat. Immunol..

[8] Zeeuwen P.L., Boekhorst J., van den Bogaard E.H., de Koning H.D., van de Kerkhof P.M., Saulnier D.M., van Swam I.I., van Hijum S.A.F.T., Kleerebezem M., Schalkwijk J. (2012). Microbiome dynamics of human epidermis following skin barrier disruption. Genome Biol..

[9] Howard B., Bascom C.C., Hu P., Binder R.L., Fadayel G., Huggins T.G., Jarrold B.B., Osborne R., Rocchetta H.L., Swift D. (2022). Aging-Associated Changes in the Adult Human Skin Microbiome and the Host Factors that Affect Skin Microbiome Composition. J. Investig. Dermatol..

[10] Kong H.H., Oh J., Deming C., Conlan S., Grice E.A., Beatson M.A., Nomicos E., Polley E.C., Komarow H.D., Nisc Comparative Sequencing Program (2012). Temporal shifts in the skin microbiome associated with disease flares and treatment in children with atopic dermatitis. Genome Res..

[11] Kretzschmar K., Watt F.M. (2014). Markers of epidermal stem cell subpopulations in adult mammalian skin. Cold Spring Harb. Perspect. Med..

[12] Lang E., Polec A., Lang A., Valk M., Blicher P., Rowe A.D., Tonseth K.A., Jackson C.J., Utheim T.P., Janssen L.M.C. (2018). Coordinated collective migration and asymmetric cell division in confluent human keratinocytes without wounding. Nat. Commun..

[13] Watt F.M. (1989). Terminal differentiation of epidermal keratinocytes. Curr. Opin. Cell Biol..

[14] Johnson J.L., Najor N.A., Green K.J. (2014). Desmosomes: Regulators of cellular signaling and adhesion in epidermal health and disease. Cold Spring Harb. Perspect. Med..

[15] Usui K., Kadono N., Furuichi Y., Shiraga K., Saitou T., Kawasaki H., Toyooka K., Tamura H., Kubo A., Amagai M. (2019). 3D in vivo imaging of the keratin filament network in the mouse stratum granulosum reveals profilaggrin-dependent regulation of keratin bundling. J. Dermatol. Sci..

[16] Monteleon C.L., Agnihotri T., Dahal A., Liu M., Rebecca V.W., Beatty G.L., Amaravadi R.K., Ridky T.W. (2018). Lysosomes Support the Degradation, Signaling, and Mitochondrial Metabolism Necessary for Human Epidermal Differentiation. J. Investig. Dermatol..

[17] Lechler T., Fuchs E. (2005). Asymmetric cell divisions promote stratification and differentiation of mammalian skin. Nature.

[18] Koria P., Andreadis S.T. (2006). Epidermal morphogenesis: The transcriptional program of human keratinocytes during stratification. J. Investig. Dermatol..

[19] Eyerich S., Eyerich K., Traidl-Hoffmann C., Biedermann T. (2018). Cutaneous Barriers and Skin Immunity: Differentiating A Connected Network. Trends Immunol..

[20] Kirschner N., Brandner J.M. (2012). Barriers and more: Functions of tight junction proteins in the skin. Ann. N. Y. Acad. Sci..

[21] Furuse M., Hata M., Furuse K., Yoshida Y., Haratake A., Sugitani Y., Noda T., Kubo A., Tsukita S. (2002). Claudin-based tight junctions are crucial for the mammalian epidermal barrier: A lesson from claudin-1-deficient mice. J. Cell Biol..

[22] Chamcheu J.C., Siddiqui I.A., Syed D.N., Adhami V.M., Liovic M., Mukhtar H. (2011). Keratin gene mutations in disorders of human skin and its appendages. Arch. Biochem. Biophys..

[23] Zhang H., Pasolli H.A., Fuchs E. (2011). Yes-associated protein (YAP) transcriptional coactivator functions in balancing growth and differentiation in skin. Proc. Natl. Acad. Sci. USA.

[24] O’Regan G.M., Sandilands A., McLean W.H.I., Irvine A.D. (2008). Filaggrin in atopic dermatitis. J. Allergy Clin. Immunol..

[25] van Smeden J., Bouwstra J.A. (2016). Stratum Corneum Lipids: Their Role for the Skin Barrier Function in Healthy Subjects and Atopic Dermatitis Patients. Curr. Probl. Dermatol..

[26] Mahanty S., Setty S.R.G. (2021). Epidermal Lamellar Body Biogenesis: Insight Into the Roles of Golgi and Lysosomes. Front. Cell Dev. Biol..

[27] Arda O., Göksügür N., Tüzün Y. (2014). Basic histological structure and functions of facial skin. Clin. Dermatol..

[28] Fluhr J.W., Kao J., Jain M., Ahn S.K., Feingold K.R., Elias P.M. (2001). Generation of free fatty acids from phospholipids regulates stratum corneum acidification and integrity. J. Investig. Dermatol..

[29] Hulpusch C., Tremmel K., Hammel G., Bhattacharyya M., de Tomassi A., Nussbaumer T., Neumann A.U., Reiger M., Traidl-Hoffmann C. (2020). Skin pH-dependent Staphylococcus aureus abundance as predictor for increasing atopic dermatitis severity. Allergy.

[30] Hachem J.P., Crumrine D., Fluhr J., Brown B.E., Feingold K.R., Elias P.M. (2003). pH directly regulates epidermal permeability barrier homeostasis, and stratum corneum integrity/cohesion. J. Investig. Dermatol..

[31] Ohkubo T., Matsumoto Y., Ogasawara Y., Sugita T. (2022). Alkaline stress inhibits the growth of Staphylococcus epidermidis by inducing TCA cycle-triggered ROS production. Biochem. Biophys. Res. Commun..

[32] Lambers H., Piessens S., Bloem A., Pronk H., Finkel P. (2006). Natural skin surface pH is on average below 5, which is beneficial for its resident flora. Int. J. Cosmet. Sci..

[33] Naik S., Bouladoux N., Linehan J.L., Han S.J., Harrison O.J., Wilhelm C., Conlan S., Himmelfarb S., Byrd A.L., Deming C. (2015). Commensal-dendritic-cell interaction specifies a unique protective skin immune signature. Nature.

[34] Wanke I., Steffen H., Christ C., Krismer B., Gotz F., Peschel A., Schaller M., Schittek B. (2011). Skin commensals amplify the innate immune response to pathogens by activation of distinct signaling pathways. J. Investig. Dermatol..

[35] Nguyen A.V., Soulika A.M. (2019). The Dynamics of the Skin’s Immune System. Int. J. Mol. Sci..

[36] Lai Y., Di Nardo A., Nakatsuji T., Leichtle A., Yang Y., Cogen A.L., Wu Z.R., Hooper L.V., Schmidt R.R., von Aulock S. (2009). Commensal bacteria regulate Toll-like receptor 3-dependent inflammation after skin injury. Nat. Med..

[37] Williams H., Campbell L., Crompton R.A., Singh G., McHugh B.J., Davidson D.J., McBain A.J., Cruickshank S.M., Hardman M.J. (2018). Microbial Host Interactions and Impaired Wound Healing in Mice and Humans: Defining a Role for BD14 and NOD2. J. Investig. Dermatol..

[38] Lebre M.C., van der Aar A.M., van Baarsen L., van Capel T.M., Schuitemaker J.H., Kapsenberg M.L., de Jong E.C. (2007). Human keratinocytes express functional Toll-like receptor 3, 4, 5, and 9. J. Investig. Dermatol..

[39] Askarian F., Wagner T., Johannessen M., Nizet V. (2018). Staphylococcus aureus modulation of innate immune responses through Toll-like (TLR), (NOD)-like (NLR) and C-type lectin (CLR) receptors. FEMS Microbiol. Rev..

[40] Zhao C., Wang I., Lehrer R.I. (1996). Widespread expression of beta-defensin hBD-1 in human secretory glands and epithelial cells. FEBS Lett..

[41] Sorensen O.E., Thapa D.R., Rosenthal A., Liu L., Roberts A.A., Ganz T. (2005). Differential regulation of beta-defensin expression in human skin by microbial stimuli. J. Immunol..

[42] Nakatsuji T., Kao M.C., Zhang L., Zouboulis C.C., Gallo R.L., Huang C.M. (2010). Sebum free fatty acids enhance the innate immune defense of human sebocytes by upregulating beta-defensin-2 expression. J. Investig. Dermatol..

[43] Dajnoki Z., Beke G., Kapitany A., Mocsai G., Gaspar K., Ruhl R., Hendrik Z., Juhasz I., Zouboulis C.C., Bacsi A. (2017). Sebaceous Gland-Rich Skin Is Characterized by TSLP Expression and Distinct Immune Surveillance Which Is Disturbed in Rosacea. J. Investig. Dermatol..

[44] Fischer C.L., Drake D.R., Dawson D.V., Blanchette D.R., Brogden K.A., Wertz P.W. (2012). Antibacterial activity of sphingoid bases and fatty acids against Gram-positive and Gram-negative bacteria. Antimicrob. Agents Chemother..

[45] Nguyen M.T., Hanzelmann D., Hartner T., Peschel A., Gotz F. (2016). Skin-Specific Unsaturated Fatty Acids Boost the Staphylococcus aureus Innate Immune Response. Infect. Immun..

[46] Beke G., Dajnoki Z., Kapitany A., Gaspar K., Medgyesi B., Poliska S., Hendrik Z., Peter Z., Torocsik D., Biro T. (2018). Immunotopographical Differences of Human Skin. Front. Immunol..

[47] Kuo I.H., Carpenter-Mendini A., Yoshida T., McGirt L.Y., Ivanov A.I., Barnes K.C., Gallo R.L., Borkowski A.W., Yamasaki K., Leung D.Y. (2013). Activation of epidermal toll-like receptor 2 enhances tight junction function: Implications for atopic dermatitis and skin barrier repair. J. Investig. Dermatol..

[48] Uberoi A., Bartow-McKenney C., Zheng Q., Flowers L., Campbell A., Knight S.A.B., Chan N., Wei M., Lovins V., Bugayev J. (2021). Commensal microbiota regulates skin barrier function and repair via signaling through the aryl hydrocarbon receptor. Cell Host Microbe.

[49] Myles I.A., Williams K.W., Reckhow J.D., Jammeh M.L., Pincus N.B., Sastalla I., Saleem D., Stone K.D., Datta S.K. (2016). Transplantation of human skin microbiota in models of atopic dermatitis. JCI Insight..

[50] Nakatsuji T., Chen T.H., Narala S., Chun K.A., Two A.M., Yun T., Shafiq F., Kotol P.F., Bouslimani A., Melnik A.V. (2017). Antimicrobials from human skin commensal bacteria protect against Staphylococcus aureus and are deficient in atopic dermatitis. Sci. Transl. Med..

[51] Vietri Rudan M., Watt F.M. (2022). Mammalian epidermis: A compendium of lipid functionality. Front. Physiol..

[52] Cleary J.M., Lipsky Z.W., Kim M., Marques C.N., German G.K. (2018). Heterogeneous ceramide distributions alter spatially resolved growth of Staphylococcus aureus on human stratum corneum. J. R. Soc. Interface..

[53] Arikawa J., Ishibashi M., Kawashima M., Takagi Y., Ichikawa Y., Imokawa G. (2002). Decreased levels of sphingosine, a natural antimicrobial agent, may be associated with vulnerability of the stratum corneum from patients with atopic dermatitis to colonization by Staphylococcus aureus. J. Investig. Dermatol..

[54] Moran J.C., Alorabi J.A., Horsburgh M.J. (2017). Comparative transcriptomics reveals discrete survival responses of *S. aureus* and *S. epidermidis* to sapienic acid. Front. Microbiol..

[55] Zheng Y., Hunt R.L., Villaruz A.E., Fisher E.L., Liu R., Liu Q., Cheung G.Y.C., Li M., Otto M. (2022). Commensal Staphylococcus epidermidis contributes to skin barrier homeostasis by generating protective ceramides. Cell Host Microbe.

[56] Sanford J.A., O’Neill A.M., Zouboulis C.C., Gallo R.L. (2019). Short-chain fatty acids from Cutibacterium acnes activate both a canonical and epigenetic inflammatory response in human sebocytes. J. Immunol..

[57] McNeil J.C., Fritz S.A. (2019). Prevention Strategies for Recurrent Community-Associated Staphylococcus aureus Skin and Soft Tissue Infections. Curr. Infect. Dis. Rep..

[58] Chen Y.E., Fischbach M.A., Belkaid Y. (2018). Skin microbiota-host interactions. Nature.

[59] Cundell A.M. (2018). Microbial Ecology of the Human Skin. Microb. Ecol..

[60] Grice E.A., Kong H.H., Conlan S., Deming C.B., Davis J., Young A.C., Bouffard G.G., Blakesley R.W., Murray P.R., Nisc Comparative Sequencing Program (2009). Topographical and Temporal Diversity of the Human Skin Microbiome. Science.

[61] Roux P.F., Oddos T., Stamatas G. (2022). Deciphering the Role of Skin Surface Microbiome in Skin Health: An Integrative Multiomics Approach Reveals Three Distinct Metabolite—Microbe Clusters. J. Investig. Dermatol..

[62] Swaney M.H., Sandstrom S., Kalan L.R. (2022). Cobamide Sharing Is Predicted in the Human Skin Microbiome. mSystems.

[63] Parlet C.P., Brown M.M., Horswill A.R. (2019). Commensal Staphylococci Influence Staphylococcus aureus Skin Colonization and Disease. Trends Microbiol..

[64] Harris T.A., Gattu S., Propheter D.C., Kuang Z., Bel S., Ruhn K.A., Chara A.L., Edwards M., Zhang C., Jo J.H. (2019). Resistin-like Molecule alpha Provides Vitamin-A-Dependent Antimicrobial Protection in the Skin. Cell Host Microbe.

[65] Lunjani N., Ahearn-Ford S., Dube F.S., Hlela C., O’Mahony L. (2021). Mechanisms of microbe-immune system dialogue within the skin. Genes Immun..

[66] Leech J.M., Dhariwala M.O., Lowe M.M., Chu K., Merana G.R., Cornuot C., Weckel A., Ma J.M., Leitner E.G., Gonzalez J.R. (2019). Toxin-Triggered Interleukin-1 Receptor Signaling Enables Early-Life Discrimination of Pathogenic versus Commensal Skin Bacteria. Cell Host Microbe.

[67] Meisel J.S., Sfyroera G., Bartow-McKenney C., Gimblet C., Bugayev J., Horwinski J., Kim B., Brestoff J.R., Tyldsley A.S., Zheng Q. (2018). Commensal microbiota modulate gene expression in the skin. Microbiome.

[68] Duncan K., Carey-Ewend K., Vaishnava S. (2021). Spatial analysis of gut microbiome reveals a distinct ecological niche associated with the mucus layer. Gut Microbes.

[69] Lu H.P., Lai Y.C., Huang S.W., Chen H.C., Hsieh C.H., Yu H.T. (2014). Spatial heterogeneity of gut microbiota reveals multiple bacterial communities with distinct characteristics. Sci. Rep..

[70] Oh J., Byrd A.L., Deming C., Conlan S., Kong H.H., Segre J.A., NISC Comparative Sequencing Program (2014). Biogeography and individuality shape function in the human skin metagenome. Nature.

[71] Costello E.K., Lauber C.L., Hamady M., Fierer N., Gordon J.I., Knight R. (2009). Bacterial community variation in human body habitats across space and time. Science.

[72] Gupta V.K., Paul S., Dutta C. (2017). Geography, Ethnicity or Subsistence-Specific Variations in Human Microbiome Composition and Diversity. Front. Microbiol..

[73] Leung M.H., Wilkins D., Lee P.K. (2015). Insights into the pan-microbiome: Skin microbial communities of Chinese individuals differ from other racial groups. Sci. Rep..

[74] Ross A.A., Doxey A.C., Neufeld J.D. (2017). The Skin Microbiome of Cohabiting Couples. mSystems.

[75] Kim H.J., Kim J.J., Myeong N.R., Kim T., Kim D., An S., Kim H., Park T., Jang S.I., Yeon J.H. (2019). Segregation of age-related skin microbiome characteristics by functionality. Sci. Rep..

[76] Staudinger T., Pipal A., Redl B. (2011). Molecular analysis of the prevalent microbiota of human male and female forehead skin compared to forearm skin and the influence of make-up. J. Appl. Microbiol..

[77] Yang Q., Liang Q., Balakrishnan B., Belobrajdic D.P., Feng Q.J., Zhang W. (2020). Role of Dietary Nutrients in the Modulation of Gut Microbiota: A Narrative Review. Nutrients.

[78] Grice E.A., Segre J.A. (2011). The skin microbiome. Nat. Rev. Microbiol..

[79] Findley K., Oh J., Yang J., Conlan S., Deming C., Meyer J.A., Schoenfeld D., Nomicos E., Park M., NIH Intramural Sequencing Center Comparative Sequencing Program (2013). Topographic diversity of fungal and bacterial communities in human skin. Nature.

[80] Zhou W., Spoto M., Hardy R., Guan C., Fleming E., Larson P.J., Brown J.S., Oh J. (2020). Host-Specific Evolutionary and Transmission Dynamics Shape the Functional Diversification of Staphylococcus epidermidis in Human Skin. Cell.

[81] Conwill A., Kuan A.C., Damerla R., Poret A.J., Baker J.S., Tripp A.D., Alm E.J., Lieberman T.D. (2022). Anatomy promotes neutral coexistence of strains in the human skin microbiome. Cell Host Microbe.

[82] Kalan L.R., Meisel J.S., Loesche M.A., Horwinski J., Soaita I., Chen X., Uberoi A., Gardner S.E., Grice E.A. (2019). Strain- and Species-Level Variation in the Microbiome of Diabetic Wounds Is Associated with Clinical Outcomes and Therapeutic Efficacy. Cell Host Microbe.

[83] Acker K.P., Wong Fok Lung T., West E., Craft J., Narechania A., Smith H., O’Brien K., Moustafa A.M., Lauren C., Planet P.J. (2019). Strains of Staphylococcus aureus that Colonize and Infect Skin Harbor Mutations in Metabolic Genes. iScience.

[84] Meric G., Mageiros L., Pensar J., Laabei M., Yahara K., Pascoe B., Kittiwan N., Tadee P., Post V., Lamble S. (2018). Disease-associated genotypes of the commensal skin bacterium Staphylococcus epidermidis. Nat. Commun..

[85] Fitz-Gibbon S., Tomida S., Chiu B.H., Nguyen L., Du C., Liu M., Elashoff D., Erfe M.C., Loncaric A., Kim J. (2013). Propionibacterium acnes strain populations in the human skin microbiome associated with acne. J. Investig. Dermatol..

[86] Mukherjee S., Mitra R., Maitra A., Gupta S., Kumaran S., Chakrabortty A., Majumder P.P. (2016). Sebum and Hydration Levels in Specific Regions of Human Face Significantly Predict the Nature and Diversity of Facial Skin Microbiome. Sci. Rep..

[87] Iyer V., Raut J., Dasgupta A. (2021). Impact of pH on growth of Staphylococcus epidermidis and Staphylococcus aureus in vitro. J. Med. Microbiol..

[88] Nakamura K., Williams M.R., Kwiecinski J.M., Horswill A.R., Gallo R.L. (2021). Staphylococcus aureus Enters Hair Follicles Using Triacylglycerol Lipases Preserved through the Genus Staphylococcus. J. Investig. Dermatol..

[89] Zhou Y., Mihindukulasuriya K.A., Gao H., La Rosa P.S., Wylie K.M., Martin J.C., Kota K., Shannon W.D., Mitreva M., Sodergren E. (2014). Exploration of bacterial community classes in major human habitats. Genome Biol..

[90] Roth R.R., James W.D. (1988). Microbial ecology of the skin. Annu. Rev. Microbiol..

[91] Hartmann A.A. (1990). The influence of various factors on the human resident skin flora. Semin. Dermatol..

[92] Otto M. (2010). Staphylococcus colonization of the skin and antimicrobial peptides. Expert Rev. Dermatol..

[93] Callewaert C., Kerckhof F.M., Granitsiotis M.S., Van Gele M., Van de Wiele T., Boon N. (2013). Characterization of Staphylococcus and Corynebacterium clusters in the human axillary region. PLoS ONE.

[94] Oh J., Byrd A.L., Park M., Kong H.H., Segre J.A. (2016). Temporal Stability of the Human Skin Microbiome. Cell.

[95] Gao Z., Perez-Perez G.I., Chen Y., Blaser M.J. (2010). Quantitation of major human cutaneous bacterial and fungal populations. J. Clin. Microbiol..

[96] Nguyen U.T., Kalan L.R. (2022). Forgotten fungi: The importance of the skin mycobiome. Curr. Opin. Microbiol..

[97] Gueho E., Midgley G., Guillot J. (1996). The genus Malassezia with description of four new species. Antonie Van Leeuwenhoek.

[98] Gupta A.K., Kohli Y., Summerbell R.C., Faergemann J. (2001). Quantitative culture of Malassezia species from different body sites of individuals with or without dermatoses. Med. Mycol..

[99] Sugita T., Suto H., Unno T., Tsuboi R., Ogawa H., Shinoda T., Nishikawa A. (2001). Molecular analysis of Malassezia microflora on the skin of atopic dermatitis patients and healthy subjects. J. Clin. Microbiol..

[100] Vierkotter A., Krutmann J. (2012). Environmental influences on skin aging and ethnic-specific manifestations. Dermatoendocrinology.

[101] Dimitriu P.A., Iker B., Malik K., Leung H., Mohn W.W., Hillebrand G.G. (2019). New Insights into the Intrinsic and Extrinsic Factors That Shape the Human Skin Microbiome. mBio.

[102] Ursell L.K., Clemente J.C., Rideout J.R., Gevers D., Caporaso J.G., Knight R. (2012). The interpersonal and intrapersonal diversity of human-associated microbiota in key body sites. J. Allergy Clin. Immunol..

[103] Ying S., Zeng D.N., Chi L., Tan Y., Galzote C., Cardona C., Lax S., Gilbert J., Quan Z.X. (2015). The Influence of Age and Gender on Skin-Associated Microbial Communities in Urban and Rural Human Populations. PLoS ONE.

[104] Kim H.J., Oh H.N., Park T., Kim H., Lee H.G., An S., Sul W.J. (2022). Aged related human skin microbiome and mycobiome in Korean women. Sci. Rep..

[105] Shibagaki N., Suda W., Clavaud C., Bastien P., Takayasu L., Iioka E., Kurokawa R., Yamashita N., Hattori Y., Shindo C. (2017). Aging-related changes in the diversity of women’s skin microbiomes associated with oral bacteria. Sci. Rep..

[106] Li Z., Bai X., Peng T., Yi X., Luo L., Yang J., Liu J., Wang Y., He T., Wang X. (2020). New Insights Into the Skin Microbial Communities and Skin Aging. Front. Microbiol..

[107] Fyhrquist N., Muirhead G., Prast-Nielsen S., Jeanmougin M., Olah P., Skoog T., Jules-Clement G., Feld M., Barrientos-Somarribas M., Sinkko H. (2019). Microbe-host interplay in atopic dermatitis and psoriasis. Nat. Commun..

[108] Thevaranjan N., Puchta A., Schulz C., Naidoo A., Szamosi J.C., Verschoor C.P., Loukov D., Schenck L.P., Jury J., Foley K.P. (2017). Age-Associated Microbial Dysbiosis Promotes Intestinal Permeability, Systemic Inflammation, and Macrophage Dysfunction. Cell Host Microbe.

[109] Perez-Munoz M.E., Arrieta M.C., Ramer-Tait A.E., Walter J. (2017). A critical assessment of the “sterile womb” and “in utero colonization” hypotheses: Implications for research on the pioneer infant microbiome. Microbiome.

[110] Gensollen T., Iyer S.S., Kasper D.L., Blumberg R.S. (2016). How colonization by microbiota in early life shapes the immune system. Science.

[111] Dominguez-Bello M.G., Costello E.K., Contreras M., Magris M., Hidalgo G., Fierer N., Knight R. (2010). Delivery mode shapes the acquisition and structure of the initial microbiota across multiple body habitats in newborns. Proc. Natl. Acad. Sci. USA.

[112] Ward T.L., Dominguez-Bello M.G., Heisel T., Al-Ghalith G., Knights D., Gale C.A. (2018). Development of the Human Mycobiome over the First Month of Life and across Body Sites. mSystems.

[113] Chu D.M., Ma J., Prince A.L., Antony K.M., Seferovic M.D., Aagaard K.M. (2017). Maturation of the infant microbiome community structure and function across multiple body sites and in relation to mode of delivery. Nat. Med..

[114] Freitas A.C., Chaban B., Bocking A., Rocco M., Yang S., Hill J.E., Money D.M., Group V.R. (2017). The vaginal microbiome of pregnant women is less rich and diverse, with lower prevalence of Mollicutes, compared to non-pregnant women. Sci. Rep..

[115] Romero R., Hassan S.S., Gajer P., Tarca A.L., Fadrosh D.W., Nikita L., Galuppi M., Lamont R.F., Chaemsaithong P., Miranda J. (2014). The composition and stability of the vaginal microbiota of normal pregnant women is different from that of non-pregnant women. Microbiome.

[116] MacIntyre D.A., Chandiramani M., Lee Y.S., Kindinger L., Smith A., Angelopoulos N., Lehne B., Arulkumaran S., Brown R., Teoh T.G. (2015). The vaginal microbiome during pregnancy and the postpartum period in a European population. Sci. Rep..

[117] Gomez de Aguero M., Ganal-Vonarburg S.C., Fuhrer T., Rupp S., Uchimura Y., Li H., Steinert A., Heikenwalder M., Hapfelmeier S., Sauer U. (2016). The maternal microbiota drives early postnatal innate immune development. Science.

[118] Scharschmidt T.C., Vasquez K.S., Truong H.A., Gearty S.V., Pauli M.L., Nosbaum A., Gratz I.K., Otto M., Moon J.J., Liese J. (2015). A Wave of Regulatory T Cells into Neonatal Skin Mediates Tolerance to Commensal Microbes. Immunity.

[119] Jensen E.T., Bertoni A.G., Crago O.L., Rotter J.I., Chen Y.I., Wood A., Rich S.S., Goodarzi M.O. (2022). Cesarean Delivery and Insulin Sensitivity in the Older Adult: The Microbiome and Insulin Longitudinal Evaluation Study. J. Endocr. Soc..

[120] Begum M., Pilkington R., Chittleborough C., Lynch J., Penno M., Smithers L. (2019). Caesarean section and risk of type 1 diabetes: Whole-of-population study. Diabet. Med..

[121] Cardwell C.R., Stene L.C., Joner G., Cinek O., Svensson J., Goldacre M.J., Parslow R.C., Pozzilli P., Brigis G., Stoyanov D. (2008). Caesarean section is associated with an increased risk of childhood-onset type 1 diabetes mellitus: A meta-analysis of observational studies. Diabetologia.

[122] Alhasan M.M., Cait A.M., Heimesaat M.M., Blaut M., Klopfleisch R., Wedel A., Conlon T.M., Yildirim A.O., Sodemann E.B., Mohn W.W. (2020). Antibiotic use during pregnancy increases offspring asthma severity in a dose-dependent manner. Allergy.

[123] Russell S.L., Gold M.J., Hartmann M., Willing B.P., Thorson L., Wlodarska M., Gill N., Blanchet M.R., Mohn W.W., McNagny K.M. (2012). Early life antibiotic-driven changes in microbiota enhance susceptibility to allergic asthma. EMBO Rep..

[124] Capone K.A., Dowd S.E., Stamatas G.N., Nikolovski J. (2011). Diversity of the human skin microbiome early in life. J. Investig. Dermatol..

[125] Zhu T., Liu X., Kong F.Q., Duan Y.Y., Yee A.L., Kim M., Galzote C., Gilbert J.A., Quan Z.X. (2019). Age and Mothers: Potent Influences of Children’s Skin Microbiota. J. Investig. Dermatol..

[126] Oh J., Conlan S., Polley E.C., Segre J.A., Kong H.H. (2012). Shifts in human skin and nares microbiota of healthy children and adults. Genome Med..

[127] Park J., Schwardt N.H., Jo J.H., Zhang Z., Pillai V., Phang S., Brady S.M., Portillo J.A., MacGibeny M.A., Liang H. (2022). Shifts in the Skin Bacterial and Fungal Communities of Healthy Children Transitioning through Puberty. J. Investig. Dermatol..

[128] Makrantonaki E., Ganceviciene R., Zouboulis C. (2011). An update on the role of the sebaceous gland in the pathogenesis of acne. Dermatoendocrinology.

[129] Schneider A.M., Nolan Z.T., Banerjee K., Paine A.R., Cong Z., Gettle S.L., Longenecker A.L., Zhan X., Agak G.W., Nelson A.M. (2023). Evolution of the facial skin microbiome during puberty in normal and acne skin. J. Eur. Acad. Dermatol. Venereol..

[130] Huang S., Haiminen N., Carrieri A.P., Hu R., Jiang L., Parida L., Russell B., Allaband C., Zarrinpar A., Vazquez-Baeza Y. (2020). Human Skin, Oral, and Gut Microbiomes Predict Chronological Age. mSystems.

[131] Yosipovitch G., Maayan-Metzger A., Merlob P., Sirota L. (2000). Skin barrier properties in different body areas in neonates. Pediatrics.

[132] Giusti F., Martella A., Bertoni L., Seidenari S. (2001). Skin barrier, hydration, and pH of the skin of infants under 2 years of age. Pediatr. Dermatol..

[133] Stamatas G.N., Nikolovski J., Luedtke M.A., Kollias N., Wiegand B.C. (2010). Infant skin microstructure assessed in vivo differs from adult skin in organization and at the cellular level. Pediatr. Dermatol..

[134] Wilantho A., Deekaew P., Srisuttiyakorn C., Tongsima S., Somboonna N. (2017). Diversity of bacterial communities on the facial skin of different age-group Thai males. PeerJ.

[135] Wilkinson H.N., Hardman M.J. (2017). The role of estrogen in cutaneous ageing and repair. Maturitas.

[136] Zouboulis C.C., Makrantonaki E. (2011). Clinical aspects and molecular diagnostics of skin aging. Clin. Dermatol..

[137] Wilkinson H.N., Hardman M.J. (2021). A role for estrogen in skin ageing and dermal biomechanics. Mech. Ageing Dev..

[138] Rabe J.H., Mamelak A.J., McElgunn P.J., Morison W.L., Sauder D.N. (2006). Photoaging: Mechanisms and repair. J. Am. Acad. Dermatol..

[139] Fang J.Y., Wang P.W., Huang C.H., Chen M.H., Wu Y.R., Pan T.L. (2016). Skin aging caused by intrinsic or extrinsic processes characterized with functional proteomics. Proteomics.

[140] Zettersten E.M., Ghadially R., Feingold K.R., Crumrine D., Elias P.M. (1997). Optimal ratios of topical stratum corneum lipids improve barrier recovery in chronologically aged skin. J. Am. Acad. Dermatol..

[141] Jugé R., Rouaud-Tinguely P., Breugnot J., Servaes K., Grimaldi C., Roth M.P., Coppin H., Closs B. (2018). Shift in skin microbiota of Western European women across aging. J. Appl. Microbiol..

[142] Nakatsuji T., Chen T.H., Butcher A.M., Trzoss L.L., Nam S.J., Shirakawa K.T., Zhou W., Oh J., Otto M., Fenical W. (2018). A commensal strain of Staphylococcus epidermidis protects against skin neoplasia. Sci. Adv..

[143] Liu H., Archer N.K., Dillen C.A., Wang Y., Ashbaugh A.G., Ortines R.V., Kao T., Lee S.K., Cai S.S., Miller R.J. (2017). Staphylococcus aureus epicutaneous exposure drives skin inflammation via IL-36-mediated T cell responses. Cell Host Microbe.

[144] Bosco N., Noti M. (2021). The aging gut microbiome and its impact on host immunity. Genes Immun..

[145] Li H., Qi Y., Jasper H. (2016). Preventing age-related decline of gut compartmentalization limits microbiota dysbiosis and extends lifespan. Cell Host Microbe.

[146] Claesen J., Spagnolo J.B., Ramos S.F., Kurita K.L., Byrd A.L., Aksenov A.A., Melnik A.V., Wong W.R., Wang S., Hernandez R.D. (2020). A Cutibacterium acnes antibiotic modulates human skin microbiota composition in hair follicles. Sci. Transl. Med..

[147] Shu M., Wang Y., Yu J., Kuo S., Coda A., Jiang Y., Gallo R.L., Huang C.M. (2013). Fermentation of Propionibacterium acnes, a commensal bacterium in the human skin microbiome, as skin probiotics against methicillin-resistant Staphylococcus aureus. PLoS ONE.

[148] O’Sullivan J.N., Rea M.C., O’Connor P.M., Hill C., Ross R.P. (2019). Human skin microbiota is a rich source of bacteriocin-producing staphylococci that kill human pathogens. FEMS Microbiol. Ecol..

[149] Zhang L., Pitcher L.E., Yousefzadeh M.J., Niedernhofer L.J., Robbins P.D., Zhu Y. (2022). Cellular senescence: A key therapeutic target in aging and diseases. J. Clin. Investig..

[150] Wilkinson H.N., Hardman M.J. (2022). Cellular Senescence in Acute and Chronic Wound Repair. Cold Spring Harb. Perspect. Biol..

[151] Chandra T., Ewels P.A., Schoenfelder S., Furlan-Magaril M., Wingett S.W., Kirschner K., Thuret J.Y., Andrews S., Fraser P., Reik W. (2015). Global reorganization of the nuclear landscape in senescent cells. Cell Rep..

[152] Kurz D.J., Decary S., Hong Y., Erusalimsky J.D. (2000). Senescence-associated (beta)-galactosidase reflects an increase in lysosomal mass during replicative ageing of human endothelial cells. J. Cell Sci..

[153] Rovira M., Sereda R., Pladevall-Morera D., Ramponi V., Marin I., Maus M., Madrigal-Matute J., Diaz A., Garcia F., Munoz J. (2022). The lysosomal proteome of senescent cells contributes to the senescence secretome. Aging Cell.

[154] Rodier F., Munoz D.P., Teachenor R., Chu V., Le O., Bhaumik D., Coppe J.P., Campeau E., Beausejour C.M., Kim S.H. (2011). DNA-SCARS: Distinct nuclear structures that sustain damage-induced senescence growth arrest and inflammatory cytokine secretion. J. Cell Sci..

[155] Perez-Mancera P.A., Young A.R., Narita M. (2014). Inside and out: The activities of senescence in cancer. Nat. Rev. Cancer.

[156] Coppé J.P., Patil C.K., Rodier F., Sun Y., Muñoz D.P., Goldstein J., Nelson P.S., Desprez P.Y., Campisi J. (2008). Senescence-associated secretory phenotypes reveal cell-nonautonomous functions of oncogenic RAS and the p53 tumor suppressor. PLoS Biol..

[157] Freund A., Patil C.K., Campisi J. (2011). p38MAPK is a novel DNA damage response-independent regulator of the senescence-associated secretory phenotype. EMBO J..

[158] Narzt M.S., Pils V., Kremslehner C., Nagelreiter I.M., Schosserer M., Bessonova E., Bayer A., Reifschneider R., Terlecki-Zaniewicz L., Waidhofer-Sollner P. (2021). Epilipidomics of Senescent Dermal Fibroblasts Identify Lysophosphatidylcholines as Pleiotropic Senescence-Associated Secretory Phenotype (SASP) Factors. J. Investig. Dermatol..

[159] Demaria M., Ohtani N., Youssef S.A., Rodier F., Toussaint W., Mitchell J.R., Laberge R.M., Vijg J., Van Steeg H., Dollé M.E. (2014). An essential role for senescent cells in optimal wound healing through secretion of PDGF-AA. Dev. Cell.

[160] Wilkinson H.N., Clowes C., Banyard K.L., Matteuci P., Mace K.A., Hardman M.J. (2019). Elevated Local Senescence in Diabetic Wound Healing Is Linked to Pathological Repair via CXCR2. J. Investig. Dermatol..

[161] Lopes-Paciencia S., Saint-Germain E., Rowell M.C., Ruiz A.F., Kalegari P., Ferbeyre G. (2019). The senescence-associated secretory phenotype and its regulation. Cytokine.

[162] Salminen A., Kauppinen A., Kaarniranta K. (2012). Emerging role of NF-kappaB signaling in the induction of senescence-associated secretory phenotype (SASP). Cell. Signal..

[163] Acosta J.C., Banito A., Wuestefeld T., Georgilis A., Janich P., Morton J.P., Athineos D., Kang T.W., Lasitschka F., Andrulis M. (2013). A complex secretory program orchestrated by the inflammasome controls paracrine senescence. Nat. Cell Biol..

[164] Victorelli S., Lagnado A., Halim J., Moore W., Talbot D., Barrett K., Chapman J., Birch J., Ogrodnik M., Meves A. (2019). Senescent human melanocytes drive skin ageing via paracrine telomere dysfunction. EMBO J..

[165] Wilkinson H.N., Hardman M.J. (2021). Wound senescence: A functional link between diabetes and ageing?. Exp. Dermatol..

[166] Waaijer M.E., Parish W.E., Strongitharm B.H., van Heemst D., Slagboom P.E., de Craen A.J., Sedivy J.M., Westendorp R.G., Gunn D.A., Maier A.B. (2012). The number of p16INK4a positive cells in human skin reflects biological age. Aging Cell.

[167] Dimri G.P., Lee X., Basile G., Acosta M., Scott G., Roskelley C., Medrano E.E., Linskens M., Rubelj I., Pereira-Smith O. (1995). A biomarker that identifies senescent human cells in culture and in aging skin in vivo. Proc. Natl. Acad. Sci. USA.

[168] Krishnamurthy J., Torrice C., Ramsey M.R., Kovalev G.I., Al-Regaiey K., Su L., Sharpless N.E. (2004). Ink4a/Arf expression is a biomarker of aging. J. Clin. Investig..

[169] Jeyapalan J.C., Ferreira M., Sedivy J.M., Herbig U. (2007). Accumulation of senescent cells in mitotic tissue of aging primates. Mech. Ageing Dev..

[170] Waldera Lupa D.M., Kalfalah F., Safferling K., Boukamp P., Poschmann G., Volpi E., Gotz-Rosch C., Bernerd F., Haag L., Huebenthal U. (2015). Characterization of Skin Aging-Associated Secreted Proteins (SAASP) Produced by Dermal Fibroblasts Isolated from Intrinsically Aged Human Skin. J. Investig. Dermatol..

[171] Prata L., Ovsyannikova I.G., Tchkonia T., Kirkland J.L. (2018). Senescent cell clearance by the immune system: Emerging therapeutic opportunities. Semin. Immunol..

[172] Munoz D.P., Yannone S.M., Daemen A., Sun Y., Vakar-Lopez F., Kawahara M., Freund A.M., Rodier F., Wu J.D., Desprez P.Y. (2019). Targetable mechanisms driving immunoevasion of persistent senescent cells link chemotherapy-resistant cancer to aging. JCI Insight.

[173] Pereira B.I., Devine O.P., Vukmanovic-Stejic M., Chambers E.S., Subramanian P., Patel N., Virasami A., Sebire N.J., Kinsler V., Valdovinos A. (2019). Senescent cells evade immune clearance via HLA-E-mediated NK and CD8(+) T cell inhibition. Nat. Commun..

[174] Le Maitre C.L., Freemont A.J., Hoyland J.A. (2007). Accelerated cellular senescence in degenerate intervertebral discs: A possible role in the pathogenesis of intervertebral disc degeneration. Arthritis Res. Ther..

[175] Parrinello S., Coppe J.P., Krtolica A., Campisi J. (2005). Stromal-epithelial interactions in aging and cancer: Senescent fibroblasts alter epithelial cell differentiation. J. Cell Sci..

[176] Dasgupta J., Kar S., Liu R., Joseph J., Kalyanaraman B., Remington S.J., Chen C., Melendez J.A. (2010). Reactive oxygen species control senescence-associated matrix metalloproteinase-1 through c-Jun-N-terminal kinase. J. Cell Physiol..

[177] Qin Z., Balimunkwe R.M., Quan T. (2017). Age-related reduction of dermal fibroblast size upregulates multiple matrix metalloproteinases as observed in aged human skin in vivo. Br. J. Dermatol..

[178] Fligiel S.E., Varani J., Datta S.C., Kang S., Fisher G.J., Voorhees J.J. (2003). Collagen degradation in aged/photodamaged skin in vivo and after exposure to matrix metalloproteinase-1 in vitro. J. Investig. Dermatol..

[179] Takaya K., Asou T., Kishi K. (2022). Aging Fibroblasts Adversely Affect Extracellular Matrix Formation via the Senescent Humoral Factor Ependymin-Related Protein 1. Cells.

[180] Shin E.-K., Park H., Noh J.-Y., Lim K.-M., Chung J.-H. (2017). Platelet Shape Changes and Cytoskeleton Dynamics as Novel Therapeutic Targets for Anti-Thrombotic Drugs. Biomol. Ther..

[181] Fisher G.J., Quan T., Purohit T., Shao Y., Cho M.K., He T., Varani J., Kang S., Voorhees J.J. (2009). Collagen fragmentation promotes oxidative stress and elevates matrix metalloproteinase-1 in fibroblasts in aged human skin. Am. J. Pathol..

[182] Choi E.J., Kil I.S., Cho E.G. (2020). Extracellular Vesicles Derived from Senescent Fibroblasts Attenuate the Dermal Effect on Keratinocyte Differentiation. Int. J. Mol. Sci..

[183] Adamus J., Aho S., Meldrum H., Bosko C., Lee J.M. (2014). p16INK4A influences the aging phenotype in the living skin equivalent. J. Investig. Dermatol..

[184] Weinmullner R., Zbiral B., Becirovic A., Stelzer E.M., Nagelreiter F., Schosserer M., Lammermann I., Liendl L., Lang M., Terlecki-Zaniewicz L. (2020). Organotypic human skin culture models constructed with senescent fibroblasts show hallmarks of skin aging. Aging Mech. Dis..

[185] Bauwens E., Paree T., Meurant S., Bouriez I., Hannart C., Wera A.C., Khelfi A., Fattaccioli A., Burteau S., Demazy C. Senescence induced by UVB in keratinocytes impairs amino acids balance. J. Investig. Dermatol..

[186] Moon K.C., Yang J.P., Lee J.S., Jeong S.H., Dhong E.S., Han S.K. (2019). Effects of Ultraviolet Irradiation on Cellular Senescence in Keratinocytes Versus Fibroblasts. J. Craniofac. Surg..

[187] Chainiaux F., Magalhaes J.P., Eliaers F., Remacle J., Toussaint O. (2002). UVB-induced premature senescence of human diploid skin fibroblasts. Int. J. Biochem. Cell Biol..

[188] Lowenau L.J., Zoschke C., Brodwolf R., Volz P., Hausmann C., Wattanapitayakul S., Boreham A., Alexiev U., Schafer-Korting M. (2017). Increased permeability of reconstructed human epidermis from UVB-irradiated keratinocytes. Eur. J. Pharm. Biopharm..

[189] Jin S.P., Han S.B., Kim Y.K., Park E.E., Doh E.J., Kim K.H., Lee D.H., Chung J.H. (2016). Changes in tight junction protein expression in intrinsic aging and photoaging in human skin in vivo. J. Dermatol. Sci..

[190] Krouwer V.J., Hekking L.H., Langelaar-Makkinje M., Regan-Klapisz E., Post J.A. (2012). Endothelial cell senescence is associated with disrupted cell-cell junctions and increased monolayer permeability. Vasc. Cell.

[191] Takahashi M., Tezuka T. (2004). The content of free amino acids in the stratum corneum is increased in senile xerosis. Arch. Dermatol. Res..

[192] Li J., Tang H., Hu X., Chen M., Xie H. (2010). Aquaporin-3 gene and protein expression in sun-protected human skin decreases with skin ageing. Australas. J. Dermatol..

[193] Jensen J.M., Forl M., Winoto-Morbach S., Seite S., Schunck M., Proksch E., Schutze S. (2005). Acid and neutral sphingomyelinase, ceramide synthase, and acid ceramidase activities in cutaneous aging. Exp. Dermatol..

[194] Pawlikowski J.S., McBryan T., van Tuyn J., Drotar M.E., Hewitt R.N., Maier A.B., King A., Blyth K., Wu H., Adams P.D. (2013). Wnt signaling potentiates nevogenesis. Proc. Natl. Acad. Sci. USA.

[195] He C., Wu Q., Hayashi N., Nakano F., Nakatsukasa E., Tsuduki T. (2020). Carbohydrate-restricted diet alters the gut microbiota, promotes senescence and shortens the life span in senescence-accelerated prone mice. J. Nutr. Biochem..

[196] Xing J., Ying Y., Mao C., Liu Y., Wang T., Zhao Q., Zhang X., Yan F., Zhang H. (2018). Hypoxia induces senescence of bone marrow mesenchymal stem cells via altered gut microbiota. Nat. Commun..

[197] Saccon T.D., Nagpal R., Yadav H., Cavalcante M.B., Nunes A.D.C., Schneider A., Gesing A., Hughes B., Yousefzadeh M., Tchkonia T. (2021). Senolytic Combination of Dasatinib and Quercetin Alleviates Intestinal Senescence and Inflammation and Modulates the Gut Microbiome in Aged Mice. J. Gerontol. A. Biol. Sci. Med. Sci..

[198] Mi W., Hu Z., Xu L., Bian X., Lian W., Yin S., Zhao S., Gao W., Guo C., Shi T. (2022). Quercetin positively affects gene expression profiles and metabolic pathway of antibiotic-treated mouse gut microbiota. Front. Microbiol..

[199] Wilmanski T., Diener C., Rappaport N., Patwardhan S., Wiedrick J., Lapidus J., Earls J.C., Zimmer A., Glusman G., Robinson M. (2021). Gut microbiome pattern reflects healthy ageing and predicts survival in humans. Nat. Metab..

[200] Andersson T., Erturk Bergdahl G., Saleh K., Magnusdottir H., Stodkilde K., Andersen C.B.F., Lundqvist K., Jensen A., Bruggemann H., Lood R. (2019). Common skin bacteria protect their host from oxidative stress through secreted antioxidant RoxP. Sci. Rep..

[201] Mathiasen S.L., Gall-Mas L., Pateras I.S., Theodorou S.D.P., Namini M.R.J., Hansen M.B., Martin O.C.B., Vadivel C.K., Ntostoglou K., Butter D. (2021). Bacterial genotoxins induce T cell senescence. Cell Rep..

[202] Wilkinson H.N., Hardman M.J. (2020). Wound healing: Cellular mechanisms and pathological outcomes. Open Biol..

[203] Gosain A., DiPietro L.A. (2004). Aging and wound healing. World J. Surg..

[204] Gould L., Abadir P., Brem H., Carter M., Conner-Kerr T., Davidson J., DiPietro L., Falanga V., Fife C., Gardner S. (2015). Chronic wound repair and healing in older adults: Current status and future research. Wound Repair Regen..

[205] Tun K., Shurko J.F., Ryan L., Lee G.C. (2018). Age-based health and economic burden of skin and soft tissue infections in the United States, 2000 and 2012. PLoS ONE.

[206] Wicke C., Bachinger A., Coerper S., Beckert S., Witte M.B., Konigsrainer A. (2009). Aging influences wound healing in patients with chronic lower extremity wounds treated in a specialized Wound Care Center. Wound Repair Regen..

[207] Guest J.F., Vowden K., Vowden P. (2017). The health economic burden that acute and chronic wounds impose on an average clinical commissioning group/health board in the UK. J. Wound Care.

[208] Nussbaum S.R., Carter M.J., Fife C.E., DaVanzo J., Haught R., Nusgart M., Cartwright D. (2018). An Economic Evaluation of the Impact, Cost, and Medicare Policy Implications of Chronic Nonhealing Wounds. Value Health.

[209] Jun J.I., Lau L.F. (2010). Cellular senescence controls fibrosis in wound healing. Aging.

[210] Sindrilaru A., Peters T., Wieschalka S., Baican C., Baican A., Peter H., Hainzl A., Schatz S., Qi Y., Schlecht A. (2011). An unrestrained proinflammatory M1 macrophage population induced by iron impairs wound healing in humans and mice. J. Clin. Investig..

[211] Mendez M.V., Stanley A., Park H.Y., Shon K., Phillips T., Menzoian J.O. (1998). Fibroblasts cultured from venous ulcers display cellular characteristics of senescence. J. Vasc. Surg..

[212] Stanley A., Osler T. (2001). Senescence and the healing rates of venous ulcers. J. Vasc. Surg..

[213] Fuentes E., Fuentes M., Alarcon M., Palomo I. (2017). Immune System Dysfunction in the Elderly. An. Acad. Bras. Cienc..

[214] Shibata K., Ogai K., Ogura K., Urai T., Aoki M., Arisandi D., Takahashi N., Okamoto S., Sanada H., Sugama J. (2021). Skin Physiology and its Microbiome as Factors Associated with the Recurrence of Pressure Injuries. Biol. Res. Nurs..

[215] Paharik A.E., Parlet C.P., Chung N., Todd D.A., Rodriguez E.I., Van Dyke M.J., Cech N.B., Horswill A.R. (2017). Coagulase-Negative Staphylococcal Strain Prevents Staphylococcus aureus Colonization and Skin Infection by Blocking Quorum Sensing. Cell Host Microbe.

[216] Rodriguez-Carlos A., Trujillo V., Gonzalez-Curiel I., Marin-Luevano S., Torres-Juarez F., Santos-Mena A., Rivas-Santiago C., Enciso-Moreno J.A., Zaga-Clavellina V., Rivas-Santiago B. (2020). Host Defense Peptide RNase 7 Is Down-regulated in the Skin of Diabetic Patients with or without Chronic Ulcers, and its Expression is Altered with Metformin. Arch. Med. Res..

[217] Redel H., Gao Z., Li H., Alekseyenko A.V., Zhou Y., Perez-Perez G.I., Weinstock G., Sodergren E., Blaser M.J. (2013). Quantitation and composition of cutaneous microbiota in diabetic and nondiabetic men. J. Infect. Dis..

[218] Kim J.H., Ruegger P.R., Lebig E.G., VanSchalkwyk S., Jeske D.R., Hsiao A., Borneman J., Martins-Green M. (2020). High Levels of Oxidative Stress Create a Microenvironment That Significantly Decreases the Diversity of the Microbiota in Diabetic Chronic Wounds and Promotes Biofilm Formation. Front. Cell. Infect. Microbiol..

[219] Wilkinson H.N., Iveson S., Catherall P., Hardman M.J. (2018). A Novel Silver Bioactive Glass Elicits Antimicrobial Efficacy Against Pseudomonas aeruginosa and Staphylococcus aureus in an ex Vivo Skin Wound Biofilm Model. Front. Microbiol..

[220] Wolcott R.D., Hanson J.D., Rees E.J., Koenig L.D., Phillips C.D., Wolcott R.A., Cox S.B., White J.S. (2016). Analysis of the chronic wound microbiota of 2,963 patients by 16S rDNA pyrosequencing. Wound Repair Regen..

[221] Colsky A.S., Kirsner R.S., Kerdel F.A. (1998). Analysis of antibiotic susceptibilities of skin wound flora in hospitalized dermatology patients. The crisis of antibiotic resistance has come to the surface. Arch. Dermatol..

[222] James G.A., Swogger E., Wolcott R., Pulcini E., Secor P., Sestrich J., Costerton J.W., Stewart P.S. (2008). Biofilms in chronic wounds. Wound Repair Regen..

[223] Siddiqui A.R., Bernstein J.M. (2010). Chronic wound infection: Facts and controversies. Clin. Dermatol..

[224] Glaudemans A.W., Uckay I., Lipsky B.A. (2015). Challenges in diagnosing infection in the diabetic foot. Diabet. Med..

[225] Pastar I., O’Neill K., Padula L., Head C.R., Burgess J.L., Chen V., Garcia D., Stojadinovic O., Hower S., Plano G.V. (2020). Staphylococcus epidermidis Boosts Innate Immune Response by Activation of Gamma Delta T Cells and Induction of Perforin-2 in Human Skin. Front. Immunol..

[226] Williams H., Crompton R.A., Thomason H.A., Campbell L., Singh G., McBain A.J., Cruickshank S.M., Hardman M.J. (2017). 2017. Cutaneous Nod2 expression regulates the skin microbiome and wound healing in a murine model. J. Investig. Dermatol..

[227] Dowd S.E., Sun Y., Secor P.R., Rhoads D.D., Wolcott B.M., James G.A., Wolcott R.D. (2008). Survey of bacterial diversity in chronic wounds using pyrosequencing, DGGE, and full ribosome shotgun sequencing. BMC Microbiol..

[228] Loesche M., Gardner S.E., Kalan L., Horwinski J., Zheng Q., Hodkinson B.P., Tyldsley A.S., Franciscus C.L., Hillis S.L., Mehta S. (2017). Temporal Stability in Chronic Wound Microbiota Is Associated With Poor Healing. J. Investig. Dermatol..

[229] MacDonald A., Brodell J.D., Daiss J.L., Schwarz E.M., Oh I. (2019). Evidence of differential microbiomes in healing versus non-healing diabetic foot ulcers prior to and following foot salvage therapy. J. Orthop. Res..

[230] Sloan T.J., Turton J.C., Tyson J., Musgrove A., Fleming V.M., Lister M.M., Loose M.W., Sockett R.E., Diggle M., Game F.L. (2019). Examining diabetic heel ulcers through an ecological lens: Microbial community dynamics associated with healing and infection. J. Med. Microbiol..

[231] Verbanic S., Shen Y., Lee J., Deacon J.M., Chen I.A. (2020). Microbial predictors of healing and short-term effect of debridement on the microbiome of chronic wounds. NPJ Biofilms Microbiomes.

[232] Gardner S.E., Hillis S.L., Heilmann K., Segre J.A., Grice E.A. (2013). The neuropathic diabetic foot ulcer microbiome is associated with clinical factors. Diabetes.

[233] Wang G., Sweren E., Liu H., Wier E., Alphonse M.P., Chen R., Islam N., Li A., Xue Y., Chen J. (2021). Bacteria induce skin regeneration via IL-1beta signaling. Cell Host Microbe.

[234] Wang G., Sweren E., Andrews W., Li Y., Chen J., Xue Y., Wier E., Alphonse M.P., Luo L., Miao Y. (2023). Commensal microbiome promotes hair follicle regeneration by inducing keratinocyte HIF-1alpha signaling and glutamine metabolism. Sci. Adv..

[235] Kong H.H., Andersson B., Clavel T., Common J.E., Jackson S.A., Olson N.D., Segre J.A., Traidl-Hoffmann C. (2017). Performing Skin Microbiome Research: A Method to the Madness. J. Investig. Dermatol..

[236] Gupta S., Mortensen M.S., Schjorring S., Trivedi U., Vestergaard G., Stokholm J., Bisgaard H., Krogfelt K.A., Sorensen S.J. (2019). Amplicon sequencing provides more accurate microbiome information in healthy children compared to culturing. Commun. Biol..

[237] Botterel F., Angebault C., Cabaret O., Stressmann F.A., Costa J.M., Wallet F., Wallaert B., Bruce K., Delhaes L. (2018). Fungal and Bacterial Diversity of Airway Microbiota in Adults with Cystic Fibrosis: Concordance Between Conventional Methods and Ultra-Deep Sequencing, and Their Practical use in the Clinical Laboratory. Mycopathologia.

[238] Mahnic A., Breznik V., Bombek Ihan M., Rupnik M. (2021). Comparison Between Cultivation and Sequencing Based Approaches for Microbiota Analysis in Swabs and Biopsies of Chronic Wounds. Front. Med..

[239] Timm C.M., Loomis K., Stone W., Mehoke T., Brensinger B., Pellicore M., Staniczenko P.P.A., Charles C., Nayak S., Karig D.K. (2020). Isolation and characterization of diverse microbial representatives from the human skin microbiome. Microbiome.

[240] Bauer E., Zimmermann J., Baldini F., Thiele I., Kaleta C. (2017). BacArena: Individual-based metabolic modeling of heterogeneous microbes in complex communities. PLoS Comput. Biol..

[241] Li L., Sohn J., Genco R.J., Wactawski-Wende J., Goodison S., Diaz P.I., Sun Y. (2022). Computational approach to modeling microbiome landscapes associated with chronic human disease progression. PLoS Comput. Biol..

[242] Woese C.R., Fox G.E. (1977). Phylogenetic structure of the prokaryotic domain: The primary kingdoms. Proc. Natl. Acad. Sci. USA.

[243] Olsen G.J., Lane D.J., Giovannoni S.J., Pace N.R., Stahl D.A. (1986). Microbial ecology and evolution: A ribosomal RNA approach. Annu. Rev. Microbiol..

[244] Brooks J.P., Edwards D.J., Harwich M.D., Rivera M.C., Fettweis J.M., Serrano M.G., Reris R.A., Sheth N.U., Huang B., Girerd P. (2015). The truth about metagenomics: Quantifying and counteracting bias in 16S rRNA studies. BMC Microbiol..

[245] Schloss P.D., Gevers D., Westcott S.L. (2011). Reducing the effects of PCR amplification and sequencing artifacts on 16S rRNA-based studies. PLoS ONE.

[246] Poulsen C.S., Ekstrøm C.T., Aarestrup F.M., Pamp S.J. (2022). Library Preparation and Sequencing Platform Introduce Bias in Metagenomic-Based Characterizations of Microbiomes. Microbiol. Spectr..

[247] Byrd A.L., Deming C., Cassidy S.K., Harrison O.J., Ng W.I., Conlan S., Belkaid Y., Segre J.A., Kong H.H., NISC Comparative Sequencing Program (2017). Staphylococcus aureus and Staphylococcus epidermidis strain diversity underlying pediatric atopic dermatitis. Sci. Transl. Med..

[248] Kong H.H. (2011). Skin microbiome: Genomics-based insights into the diversity and role of skin microbes. Trends Mol. Med..

[249] De Filippis F., Laiola M., Blaiotta G., Ercolini D. (2017). Different Amplicon Targets for Sequencing-Based Studies of Fungal Diversity. Appl. Environ. Microbiol..

[250] Marizzoni M., Gurry T., Provasi S., Greub G., Lopizzo N., Ribaldi F., Festari C., Mazzelli M., Mombelli E., Salvatore M. (2020). Comparison of Bioinformatics Pipelines and Operating Systems for the Analyses of 16S rRNA Gene Amplicon Sequences in Human Fecal Samples. Front. Microbiol..

[251] Malla M.A., Dubey A., Kumar A., Yadav S., Hashem A., Abd_Allah E.F. (2018). Exploring the Human Microbiome: The Potential Future Role of Next-Generation Sequencing in Disease Diagnosis and Treatment. Front. Immunol..

[252] Pearman W.S., Freed N.E., Silander O.K. (2020). Testing the advantages and disadvantages of short- and long- read eukaryotic metagenomics using simulated reads. BMC Bioinform..

[253] McLaren M.R., Willis A.D., Callahan B.J. (2019). Consistent and correctable bias in metagenomic sequencing experiments. eLife.

[254] Acosta E.M., Little K.A., Bratton B.P., Mao X., Payne A., Devenport D., Gitai Z. (2021). Bacterial DNA on the skin surface overrepresents the viable skin microbiome. BioRxiv.

[255] Chen L., Zhao N., Cao J., Liu X., Xu J., Ma Y., Yu Y., Zhang X., Zhang W., Guan X. (2022). Short-and long-read metagenomics expand individualized structural variations in gut microbiomes. Nat Commun..

[256] Xia Y., Li X., Wu Z., Nie C., Cheng Z., Sun Y., Liu L., Zhang T. (2023). Strategies and tools in illumina and nanopore-integrated metagenomic analysis of microbiome data. iMeta.

[257] Spittaels K.J., Ongena R., Zouboulis C.C., Crabbe A., Coenye T. (2020). Cutibacterium acnes Phylotype I and II Strains Interact Differently With Human Skin Cells. Front. Cell Infect. Microbiol..

[258] Yu Y., Champer J., Agak G.W., Kao S., Modlin R.L., Kim J. (2016). Different Propionibacterium acnes Phylotypes Induce Distinct Immune Responses and Express Unique Surface and Secreted Proteomes. J. Investig. Dermatol..

[259] Saheb Kashaf S., Proctor D.M., Deming C., Saary P., Holzer M., Taylor M.E., Kong H.H., Segre J.A., Almeida A., NISC Comparative Sequencing Program (2022). Integrating cultivation and metagenomics for a multi-kingdom view of skin microbiome diversity and functions. Nat. Microbiol..

[260] Thingholm L.B., Ruhlemann M.C., Koch M., Fuqua B., Laucke G., Boehm R., Bang C., Franzosa E.A., Hubenthal M., Rahnavard A. (2019). Obese Individuals with and without Type 2 Diabetes Show Different Gut Microbial Functional Capacity and Composition. Cell Host Microbe.

[261] Zheng W., Zhao S., Yin Y., Zhang H., Needham D.M., Evans E.D., Dai C.L., Lu P.J., Alm E.J., Weitz D.A. (2022). High-throughput, single-microbe genomics with strain resolution, applied to a human gut microbiome. Science.

[262] Gotschlich E.C., Colbert R.A., Gill T. (2019). Methods in microbiome research: Past, present, and future. Best. Pract. Res. Clin. Rheumatol..

[263] Ruiz-Perez C.A., Conrad R.E., Konstantinidis K.T. (2021). MicrobeAnnotator: A user-friendly, comprehensive functional annotation pipeline for microbial genomes. BMC Bioinform..

[264] Wemheuer F., Taylor J.A., Daniel R., Johnston E., Meinicke P., Thomas T., Wemheuer B. (2020). Tax4Fun2: Prediction of habitat-specific functional profiles and functional redundancy based on 16S rRNA gene sequences. Environ. Microbiome.

[265] Parks D.H., Tyson G.W., Hugenholtz P., Beiko R.G. (2014). STAMP: Statistical analysis of taxonomic and functional profiles. Bioinformatics.

[266] Martin S., Heavens D., Lan Y., Horsfield S., Clark M.D., Leggett R.M. (2022). Nanopore adaptive sampling: A tool for enrichment of low abundance species in metagenomic samples. Genome Biol..

[267] Sinha R., Abu-Ali G., Vogtmann E., Fodor A.A., Ren B., Amir A., Schwager E., Crabtree J., Ma S., The Microbiome Quality Control Project Consortium (2017). Assessment of variation in microbial community amplicon sequencing by the Microbiome Quality Control (MBQC) project consortium. Nat. Biotechnol..

[268] Peabody M.A., Van Rossum T., Lo R., Brinkman F.S. (2015). Evaluation of shotgun metagenomics sequence classification methods using in silico and in vitro simulated communities. BMC Bioinformatics.

[269] Wilkinson H.N., Guinn B.A., Hardman M.J. (2021). Combined Metallomics/Transcriptomics Profiling Reveals a Major Role for Metals in Wound Repair. Front. Cell. Dev. Biol..

[270] Belkaid Y., Harrison O.J. (2017). Homeostatic Immunity and the Microbiota. Immunity.

[271] Larson P.J., Chong D., Fleming E., Oh J. (2021). Challenges in Developing a Human Model System for Skin Microbiome Research. J. Investig. Dermatol..

[272] Kohda K., Li X., Soga N., Nagura R., Duerna T., Nakajima S., Nakagawa I., Ito M., Ikeuchi A. (2021). An In Vitro Mixed Infection Model With Commensal and Pathogenic Staphylococci for the Exploration of Interspecific Interactions and Their Impacts on Skin Physiology. Front. Cell. Infect. Microbiol..

[273] Wilkinson H.N., McBain A.J., Stephenson C., Hardman M.J. (2016). Comparing the Effectiveness of Polymer Debriding Devices Using a Porcine Wound Biofilm Model. Adv. Wound Care..

[274] Popov L., Kovalski J., Grandi G., Bagnoli F., Amieva M.R. (2014). Three-Dimensional Human Skin Models to Understand Staphylococcus aureus Skin Colonization and Infection. Front. Immunol..

[275] Di Grazia A., Luca V., Segev-Zarko L.A., Shai Y., Mangoni M.L. (2014). Temporins A and B stimulate migration of HaCaT keratinocytes and kill intracellular Staphylococcus aureus. Antimicrob. Agents Chemother..

[276] Williams M.R., Nakatsuji T., Sanford J.A., Vrbanac A.F., Gallo R.L. (2017). Staphylococcus aureus Induces Increased Serine Protease Activity in Keratinocytes. J. Investig. Dermatol..

[277] Rademacher F., Simanski M., Hesse B., Dombrowsky G., Vent N., Glaser R., Harder J. (2019). Staphylococcus epidermidis Activates Aryl Hydrocarbon Receptor Signaling in Human Keratinocytes: Implications for Cutaneous Defense. J. Innate Immun..

[278] McKee T.J., Komarova S.V. (2017). Is it time to reinvent basic cell culture medium?. Am. J. Physiol. Cell Physiol..

[279] Viano M., Alotto D., Aillon A., Castagnoli C., Silvagno F. (2017). A thermal gradient modulates the oxidative metabolism and growth of human keratinocytes. FEBS Open Bio..

[280] van der Krieken D.A., Ederveen T.H., van Hijum S.A., Jansen P.A., Melchers W.J., Scheepers P.T., Schalkwijk J., Zeeuwen P.L. (2016). An In vitro Model for Bacterial Growth on Human Stratum Corneum. Acta Derm. Venereol..

[281] Clement V., Roy V., Pare B., Goulet C.R., Deschenes L.T., Berthod F., Bolduc S., Gros-Louis F. (2022). Tridimensional cell culture of dermal fibroblasts promotes exosome-mediated secretion of extracellular matrix proteins. Sci. Rep..

[282] Mieremet A., Rietveld M., Absalah S., van Smeden J., Bouwstra J.A., El Ghalbzouri A. (2017). Improved epidermal barrier formation in human skin models by chitosan modulated dermal matrices. PLoS ONE.

[283] van Drongelen V., Haisma E.M., Out-Luiting J.J., Nibbering P.H., El Ghalbzouri A. (2014). Reduced filaggrin expression is accompanied by increased Staphylococcus aureus colonization of epidermal skin models. Clin. Exp. Allergy.

[284] Zhang Z., Michniak-Kohn B.B. (2012). Tissue engineered human skin equivalents. Pharmaceutics.

[285] Loomis K.H., Wu S.K., Ernlund A., Zudock K., Reno A., Blount K., Karig D.K. (2021). A mixed community of skin microbiome representatives influences cutaneous processes more than individual members. Microbiome.

[286] Lemoine L., Dieckmann R., Al Dahouk S., Vincze S., Luch A., Tralau T. (2020). Microbially competent 3D skin: A test system that reveals insight into host-microbe interactions and their potential toxicological impact. Arch. Toxicol..

[287] Abaci H.E., Guo Z., Coffman A., Gillette B., Lee W.H., Sia S.K., Christiano A.M. (2016). Human Skin Constructs with Spatially Controlled Vasculature Using Primary and iPSC-Derived Endothelial Cells. Adv. Healthc. Mater..

[288] Abaci H.E., Coffman A., Doucet Y., Chen J., Jackow J., Wang E., Guo Z., Shin J.U., Jahoda C.A., Christiano A.M. (2018). Tissue engineering of human hair follicles using a biomimetic developmental approach. Nat. Commun..

[289] Griffoni C., Neidhart B., Yang K., Groeber-Becker F., Maniura-Weber K., Dandekar T., Walles H., Rottmar M. (2021). In vitro skin culture media influence the viability and inflammatory response of primary macrophages. Sci. Rep..

[290] Shin J.U., Abaci H.E., Herron L., Guo Z., Sallee B., Pappalardo A., Jackow J., Wang E.H.C., Doucet Y., Christiano A.M. (2020). Recapitulating T cell infiltration in 3D psoriatic skin models for patient-specific drug testing. Sci. Rep..

[291] Rakita A., Nikolic N., Mildner M., Matiasek J., Elbe-Burger A. (2020). Re-epithelialization and immune cell behaviour in an ex vivo human skin model. Sci. Rep..

[292] Wilkinson H.N., Kidd A.S., Roberts E.R., Hardman M.J. (2021). Human Ex vivo Wound Model and Whole-Mount Staining Approach to Accurately Evaluate Skin Repair. J. Vis. Exp..

[293] Lebonvallet N., Jeanmaire C., Danoux L., Sibille P., Pauly G., Misery L. (2010). The evolution and use of skin explants: Potential and limitations for dermatological research. Eur. J. Dermatol..

[294] Choi E.H. (2018). Gender, Age, and Ethnicity as Factors That Can Influence Skin pH. Curr. Probl. Dermatol..

[295] Larouche J., Sheoran S., Maruyama K., Martino M.M. (2018). Immune Regulation of Skin Wound Healing: Mechanisms and Novel Therapeutic Targets. Adv. Wound Care..

[296] Zimmermann C., Troeltzsch D., Gimenez-Rivera V.A., Galli S.J., Metz M., Maurer M., Siebenhaar F. (2019). Mast cells are critical for controlling the bacterial burden and the healing of infected wounds. Proc. Natl. Acad. Sci. USA.

[297] Atac B., Wagner I., Horland R., Lauster R., Marx U., Tonevitsky A.G., Azar R.P., Lindner G. (2013). Skin and hair on-a-chip: In vitro skin models versus ex vivo tissue maintenance with dynamic perfusion. Lab Chip..

[298] Ramadan Q., Ting F.C. (2016). In vitro micro-physiological immune-competent model of the human skin. Lab Chip..

[299] Linehan J.L., Harrison O.J., Han S.J., Byrd A.L., Vujkovic-Cvijin I., Villarino A.V., Sen S.K., Shaik J., Smelkinson M., Tamoutounour S. (2018). Non-classical Immunity Controls Microbiota Impact on Skin Immunity and Tissue Repair. Cell.

[300] Scharschmidt T.C., Vasquez K.S., Pauli M.L., Leitner E.G., Chu K., Truong H.A., Lowe M.M., Sanchez Rodriguez R., Ali N., Laszik Z.G. (2017). Commensal Microbes and Hair Follicle Morphogenesis Coordinately Drive Treg Migration into Neonatal Skin. Cell Host Microbe.

[301] Ovchinnikov K.V., Kranjec C., Thorstensen T., Carlsen H., Diep D.B. (2020). Successful Development of Bacteriocins into Therapeutic Formulation for Treatment of MRSA Skin Infection in a Murine Model. Antimicrob. Agents Chemother..

[302] Eichenseher F., Herpers B.L., Badoux P., Leyva-Castillo J.M., Geha R.S., van der Zwart M., McKellar J., Janssen F., de Rooij B., Selvakumar L. (2022). Linker-Improved Chimeric Endolysin Selectively Kills Staphylococcus aureus In Vitro, on Reconstituted Human Epidermis, and in a Murine Model of Skin Infection. Antimicrob. Agents Chemother..

[303] Shay T., Jojic V., Zuk O., Rothamel K., Puyraimond-Zemmour D., Feng T., Wakamatsu E., Benoist C., Koller D., Regev A. (2013). Conservation and divergence in the transcriptional programs of the human and mouse immune systems. Proc. Natl. Acad. Sci. USA.

[304] Zomer H.D., Trentin A.G. (2018). Skin wound healing in humans and mice: Challenges in translational research. J. Dermatol. Sci..

[305] Youn C., Archer N.K., Miller L.S. (2020). Research Techniques Made Simple: Mouse Bacterial Skin Infection Models for Immunity Research. J. Investig. Dermatol..

[306] Xiao L., Feng Q., Liang S., Sonne S.B., Xia Z., Qiu X., Li X., Long H., Zhang J., Zhang D. (2015). A catalog of the mouse gut metagenome. Nat. Biotechnol..

[307] Campbell J.H., Foster C.M., Vishnivetskaya T., Campbell A.G., Yang Z.K., Wymore A., Palumbo A.V., Chesler E.J., Podar M. (2012). Host genetic and environmental effects on mouse intestinal microbiota. ISME J..

[308] Percie du Sert N., Ahluwalia A., Alam S., Avey M.T., Baker M., Browne W.J., Clark A., Cuthill I.C., Dirnagl U., Emerson M. (2020). Reporting animal research: Explanation and elaboration for the ARRIVE guidelines 2.0. PLoS Biol..

[309] Summerfield A., Meurens F., Ricklin M.E. (2015). The immunology of the porcine skin and its value as a model for human skin. Mol. Immunol..

[310] O’Neill A.M., Nakatsuji T., Hayachi A., Williams M.R., Mills R.H., Gonzalez D.J., Gallo R.L. (2020). Identification of a Human Skin Commensal Bacterium that Selectively Kills Cutibacterium acnes. J. Investig. Dermatol..

[311] Ovchinnikov K.V., Kranjec C., Telke A., Kjos M., Thorstensen T., Scherer S., Carlsen H., Diep D.B. (2021). A Strong Synergy Between the Thiopeptide Bacteriocin Micrococcin P1 and Rifampicin Against MRSA in a Murine Skin Infection Model. Front. Immunol..

[312] Myles I.A., Earland N.J., Anderson E.D., Moore I.N., Kieh M.D., Williams K.W., Saleem A., Fontecilla N.M., Welch P.A., Darnell D.A. (2018). First-in-human topical microbiome transplantation with Roseomonas mucosa for atopic dermatitis. JCI Insight..

[313] Ni Y., Yang X., Zheng L., Wang Z., Wu L., Jiang J., Yang T., Ma L., Fu Z. (2019). Lactobacillus and Bifidobacterium Improves Physiological Function and Cognitive Ability in Aged Mice by the Regulation of Gut Microbiota. Mol. Nutr. Food Res..

[314] Huang S.Y., Chen L.H., Wang M.F., Hsu C.C., Chan C.H., Li J.X., Huang H.Y. (2018). Lactobacillus paracasei PS23 Delays Progression of Age-Related Cognitive Decline in Senescence Accelerated Mouse Prone 8 (SAMP8) Mice. Nutrients.

[315] Morita Y., Jounai K., Sakamoto A., Tomita Y., Sugihara Y., Suzuki H., Ohshio K., Otake M., Fujiwara D., Kanauchi O. (2018). Long-term intake of Lactobacillus paracasei KW3110 prevents age-related chronic inflammation and retinal cell loss in physiologically aged mice. Aging.

[316] Nam Y., Kim J., Baek J., Kim W. (2021). Improvement of Cutaneous Wound Healing via Topical Application of Heat-Killed Lactococcus chungangensis CAU 1447 on Diabetic Mice. Nutrients.

[317] Mohtashami M., Mohamadi M., Azimi-Nezhad M., Saeidi J., Nia F.F., Ghasemi A. (2021). Lactobacillus bulgaricus and Lactobacillus plantarum improve diabetic wound healing through modulating inflammatory factors. Biotechnol. Appl. Biochem..

[318] Hernando-Amado S., Coque T.M., Baquero F., Martinez J.L. (2019). Defining and combating antibiotic resistance from One Health and Global Health perspectives. Nat. Microbiol..

[319] Josefsdottir K.S., Baldridge M.T., Kadmon C.S., King K.Y. (2017). Antibiotics impair murine hematopoiesis by depleting the intestinal microbiota. Blood.

[320] de Nies L., Busi S.B., Tsenkova M., Halder R., Letellier E., Wilmes P. (2022). Evolution of the murine gut resistome following broad-spectrum antibiotic treatment. Nat. Commun..

